# *ACLY* alternative splicing correlates with cancer phenotypes

**DOI:** 10.1016/j.jbc.2024.107418

**Published:** 2024-05-28

**Authors:** Julianna G. Supplee, Hayley C. Affronti, Richard Duan, Rebekah C. Brooks, Zachary E. Stine, Phuong T.T. Nguyen, Laura V. Pinheiro, Michael C. Noji, Jack M. Drummond, Kevin Huang, Kollin Schultz, Chi V. Dang, Ronen Marmorstein, Kathryn E. Wellen

**Affiliations:** 1 Department of Cancer Biology, University of Pennsylvania, Philadelphia, Pennsylvania, USA; 2 Department of Biochemistry and Biophysics, University of Pennsylvania, Philadelphia, Pennsylvania, USA; 3 The Wistar Institute, Philadelphia, Pennsylvania, USA; 4 Department of Oncology, Johns Hopkins University School of Medicine, Baltimore, Maryland, USA; 5 The Ludwig Institute for Cancer Research, New York, New York, USA

**Keywords:** Alternative splicing, ATP-citrate lyase, Cancer, Epithelial Splicing Regulatory Protein 1, Isoforms, Metabolism, TCGA

## Abstract

ATP-citrate lyase (ACLY) links carbohydrate and lipid metabolism and provides nucleocytosolic acetyl-CoA for protein acetylation. *ACLY* has two major splice isoforms: the full-length canonical “long” isoform and an uncharacterized “short” isoform in which exon 14 is spliced out. Exon 14 encodes 10 amino acids within an intrinsically disordered region and includes at least one dynamically phosphorylated residue. Both isoforms are expressed in healthy tissues to varying degrees. Analysis of human transcriptomic data revealed that the percent spliced in (PSI) of exon 14 is increased in several cancers and correlated with poorer overall survival in a pan-cancer analysis, though not in individual tumor types. This prompted us to explore potential biochemical and functional differences between ACLY isoforms. Here, we show that there are no discernible differences in enzymatic activity or stability between isoforms or phosphomutants of ACLY *in vitro*. Similarly, both isoforms and phosphomutants were able to rescue ACLY functions, including fatty acid synthesis and bulk histone acetylation, when re-expressed in *Acly* knockout cells. Deletion of *Acly* exon 14 in mice did not overtly impact development or metabolic physiology nor did it attenuate tumor burden in a genetic model of intestinal cancer. Notably, expression of epithelial splicing regulatory protein 1 (*ESRP1*) is highly correlated with *ACLY* PSI. We report that *ACLY* splicing is regulated by *ESRP1*. In turn, both *ESRP1* expression and *ACLY* PSI are correlated with specific immune signatures in tumors. Despite these intriguing patterns of *ACLY* splicing in healthy and cancer tissues, functional differences between the isoforms remain elusive.

First noted by Otto Warburg almost 100 years ago, cancer cells have an altered metabolic phenotype compared to healthy cells (1). To survive and proliferate, tumor cells must synthesize macromolecules and maintain energy and redox balance under conditions that are often nutrient- and oxygen-poor, requiring coordinated reprogramming of cellular metabolism. One mechanism to achieve this favorable reprogramming is alternative splicing of metabolic enzymes. RNA splicing is a tightly controlled process; in cancer, aberrant splicing consistently occurs in ways that promote transformation (2). Specifically, enzymes which are considered as metabolic “gatekeepers” are often alternatively spliced in cancer, such as glutaminase in glutamine metabolism (3), ketohexokinase in fructose metabolism (4), and pyruvate kinase in glycolysis (5). A particularly relevant example is the PKM2 isoform of pyruvate kinase, which is preferentially expressed in cancer and other proliferating cells (5). Compared to PKM1, the PKM2 isoform has altered biochemical activity, undergoes different post-translational modifications, and may translocate to the nucleus and interact with specific transcription factors, all of which modulate glycolytic flux to support proliferation and tumorigenesis (5, 6, 7, 8). Small molecules that modulate PKM2 activity to resemble PKM1 can change the metabolic profile of cancer cells in culture and inhibit growth of tumors *in vivo*, demonstrating the potential utility of isoform-specific therapies (9). While targeting metabolic enzymes to treat cancer is gaining traction (10), the biological and therapeutic roles of specific splice isoforms are often overlooked.

ATP-citrate lyase (ACLY) catalyzes the cleavage of extramitochondrial citrate into acetyl-CoA and oxaloacetate, thus providing a major source of nucleocytosolic acetyl-CoA in the cell. In the cytosol, acetyl-CoA is used for lipid synthesis, while the availability of acetyl-CoA in the nucleus corresponds to histone acetylation and subsequent changes in gene expression (11). ACLY has been investigated as a potential therapeutic target for metabolic diseases and cancer, and several ACLY inhibitors have been developed (12, 13, 14, 15). Bempedoic acid inhibits hepatic ACLY and is FDA approved to treat hypercholesterolemia and has been shown to reduce adverse cardiac events (16). *ACLY* is overexpressed in several tumor types (17, 18, 19), and inhibition or knockout (KO) impairs lipid synthesis and tumor growth *in vivo* (19, 20, 21, 22, 23).

ACLY is a homotetramer with an intrinsically disordered region (IDR) which is unresolved in the cryo-electron microscopy structure of the intact human protein (24), but its location can be approximated using AlphaFold (Fig. 1*A*). This IDR spans amino acids 429 to 493 within the human and mouse sequences and includes 10 amino acids encoded by the cassette exon 14 (Fig. 1*B*). The IDR is host to several known regulatory phosphorylation sites. Serine 455 is phosphorylated by AKT and PKA in response to insulin stimulation (25), DNA damage (26), and during brown fat adipogenesis (27) and is a prerequisite for phosphorylation of threonine 477 and serine 451 by GSK3β (28). Within the exon 14-encoded region, serine 481 phosphorylation has been detected in several phosphoproteomics studies in the context of the cell cycle (29, 30, 31), cancer (32, 33), differentiation (34), and various signaling pathways (35, 36, 37). While the IDR, including exon 14, is well-conserved in mammals, it is markedly less conserved in other vertebrates (Fig. 1*A*) and quite variable in invertebrates (not shown), suggesting its functions are specific to mammalian physiology.

**Figure 1:**
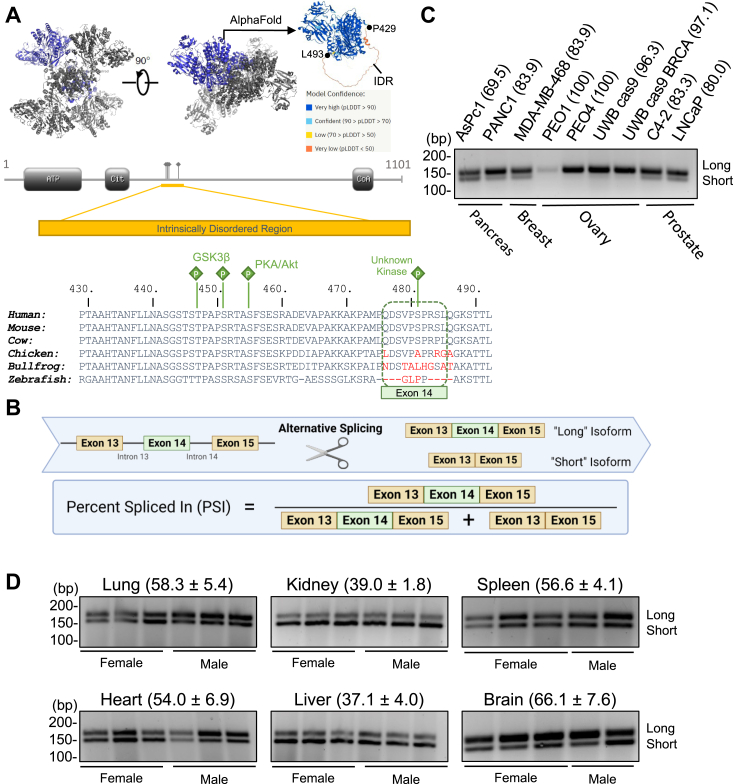
**A “short” ACLY isoform is expressed in human and mouse tissues.***A*, structure of ACLY (PDB: 6UUZ) and schematic of the intrinstically disordered region (IDR) including sequence alignment. Residues flanking the IDR are indicated with *black dots*. *B*, schematics of ACLY splicing and percent spliced in (PSI) of ACLY exon 14. *C*, RT-PCR of ACLY splice isoforms in human cancer cell lines. PSI is reported in parentheses. *D*, RT-PCR of ACLY splice isoforms in tissues from *ad lib* fed 10- to 12-week-old C57BL/6J mice. Average and standard deviation of PSI for each organ is reported in parentheses. ACLY, ATP-citrate lyase.

In this study, we evaluated the functional differences of the ACLY isoforms with (long) and without (short) exon 14 in mice and in cells and their biochemical activities *in vitro*. Analysis of human transcriptomic data revealed that the proportion of long isoform is increased in several cancers and correlated with poorer overall survival in a pan-cancer analysis. Despite this correlation, the two ACLY isoforms did not show detectable differences in enzymatic activity or stability *in vitro* or phenotypic differences in cells or mice. However, we found that the ACLY splicing pattern is correlated with the expression of epithelial splicing regulatory protein 1 (*ESRP1*) and that ACLY splicing is regulated by ESRP1. Together, these data reveal the expression of two ACLY splice isoforms in mouse and human tissues and indicate that ACLY is a component of the splicing program mediated by ESRP1, a factor that has been implicated in tumor progression.

## Results

### A “short” ACLY isoform is expressed in human and mouse

To investigate the potential for *ACLY* splicing regulation across tissues, we examined the Vertebrate Alternative Splicing and Transcription Database (VastDB) (38). Four alternative splicing events are reported in addition to exon 14 skipping; two do not impact the protein-coding region (both in 5′ UTR) and the remaining two cause a frameshift that results in a truncated, nonfunctional protein. Regarding exon 14, both long and short *ACLY* isoforms are expressed across human tissues, with relatively low percent spliced in (PSI, see Fig. 1*B*) in kidney (43%) and much higher PSI in whole brain (82%) (Fig. S1*A*). To detect *ACLY* isoforms in cells and tissues, RT-PCR primers were designed to flank exon 14 (Fig. S1*B*). The PCR products from the isoforms could then be resolved on an agarose gel (Fig. S1*C*). Both isoforms, though primarily the long isoform, were detected in a panel of human cancer cell lines (Fig. 1*C*). Consistent with human transcripts reported in VastDB, the long and short isoforms were clearly detected in several normal mouse tissues, with kidney and liver having the lowest *Acly* PSI and brain having the highest PSI among tissues assayed in both males and females (Fig. 1*D*). Additionally, there do not appear to be sex-based differences in ACLY isoform expression.

### High *ACLY* PSI across cancers is associated with poor patient outcomes

**Figure 2:**
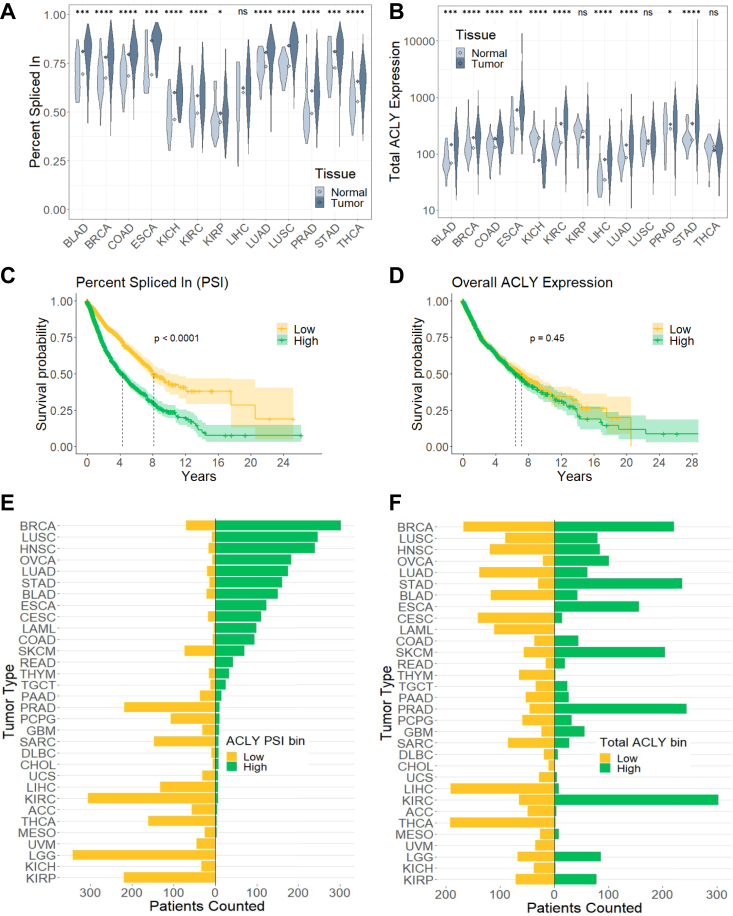
**High ACLY PSI across cancers is associated with poor patient outcomes.***A* and *B*, comparison of ACLY (*A*) PSI and (*B*) total expression in normal and tumor samples from TCGA. Groups were compared using two-tailed *t* test. ns (*p* > 0.05), *p* ≤ 0.05 (∗), *p* ≤ 0.001 (∗∗∗), and *p* ≤ 0.0001 (∗∗∗∗). *C* and *D*, Kaplan–Meyer survival curves of TCGA patients stratified by *upper* (*high*) and *lower* (*low*) quartile ACLY (*C*) PSI and (*D*) total expression, where *dotted lines* indicated time at 0.50 survival probability. Groups were compared using log-rank test. *E* and *F*, binning of TCGA patients into high and low ACLY (*E*) PSI and (*F*) total expression. ACLY, ATP-citrate lyase; PSI, percent spliced in; TCGA, The Cancer Genome Atlas.

Since ACLY has been shown to support tumor growth, we used data from The Cancer Genome Atlas (TCGA) to form hypotheses about the function of *ACLY* splice isoforms. Both *ACLY* PSI and overall expression were consistently elevated across most tumor types compared to normal tissue (Fig. 2, *A* and *B*). No clear differences in *ACLY* PSI were observed with the stage of the tumor, suggesting that this splicing shift may be an early event in tumorigenesis (Fig. S2*A*). When all tumors are stratified by high PSI (upper quartile) and low PSI (lower quartile), patients with high PSI tumors have worse survival outcomes (Fig. 2*C*). This trend is not seen when a similar analysis is done by stratifying based on overall *ACLY* expression (Fig. 2*D*). Looking at each isoform individually, most tumor types tend to have upregulated long isoform compared to normal tissue, while the short isoform is usually unchanged or in some cases downregulated (Fig. S2, *B* and *D*), suggesting that the increased *ACLY* PSI seen in tumors is mostly driven by the long isoform. Survival analysis of patients stratified by expression of individual isoforms echoed the trend of high PSI correlation with worse outcomes (Fig. S2, *C* and *E*), but PSI stratification showed the greatest difference (Fig. 2*C*). Therefore, we continued to use PSI as our primary metric of *ACLY* splicing. Together the data indicate that *ACLY* PSI increases across multiple cancer types, and when considering all cancers, higher PSI correlates with poorer survival.

We next asked whether the relationship between PSI and patient survival held within specific tumor types, finding that the correlation between PSI and survival is not apparent when looking at individual tumor types (Fig. S2*F*). Within a particular tumor type, cases have almost exclusively either high *ACLY* PSI (*e.g.*, lung, ovarian, and colon) or low *ACLY* PSI (*e.g.*, prostate, liver, and kidney) (Fig. 2*E*). In contrast, total *ACLY* expression is much more variable (Fig. 2*F*). In other words, there is little variation in PSI within each tumor type, which may explain why analyzing *ACLY* splicing in individual cancers does not yield similar trends as a pan-cancer analysis. Alternatively, the trends observed in the pan-cancer analysis might simply reflect that cancers with low rates of patient survival are those with high *ACLY* PSI.

### ACLY isoforms and phosphomutants have similar biochemical properties *in vitro* and rescue functions comparably in *Acly* KO cells

**Figure 3:**
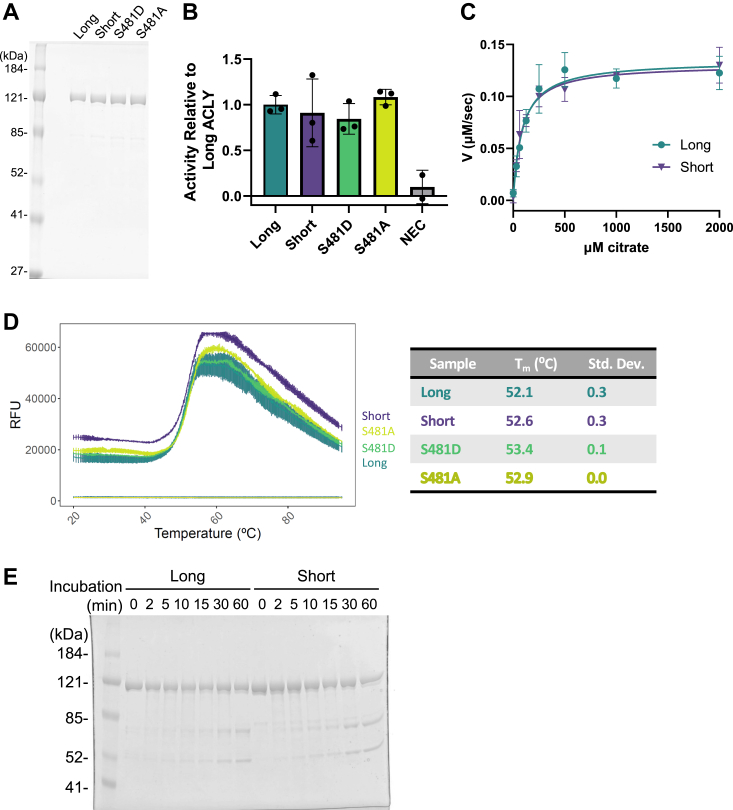
**ACLY isoforms and phosphomutants have similar properties *in vitro*.***A*, coomassie blue-stained SDS-PAGE of ACLY purified from *E. coli*. *B*, relative activity of purified ACLY using an MDH-linked assay. Data points represent technical replicates. *C*, Michaelis–Menten kinetics of long and short ACLY with respect to citrate. Data points represent technical replicates. *D*, differential scanning fluorimetry melt curves of ACLY in triplicate (*left*). Calculated T_m_ from sigmoid fitting (*right*). Two-tailed *t* tests between each pair was not significant (*p* > 0.05). *E*, Coomassie-stained SDS-PAGE of long and short ACLY after digestion with 1:50 M equivalent of chymotrypsin for indicated time points. ACLY, ATP-citrate lyase; NEC, no enzyme control.

We next investigated whether ACLY isoforms possess functional differences. First, to determine if exon 14 inclusion impacted ACLY enzymatic activity, human recombinant long, short, S481D (phosphomimic) and S481A (phosphorylation preventing) ACLY species were purified from *E. coli* (Fig. 3*A*) and their relative catalytic activities and stabilities evaluated. Each protein had similar relative catalytic rates (Fig. 3*B*), and the isoforms had nearly identical Michaelis–Menten kinetics with respect to citrate (Fig. 3*C*). After curve fitting using GraphPad Prism, the K_m_ for the long and short isoforms was 88.0 μM [60.8, 124.8] and 80.9 μM [57.8, 111.5]; k_cat_ was 3.36 s^−1^ [3.07, 3.67] and 3.26 s^−1^ [3.02, 3.53]; and k_cat_/K_m_ was 0.0381 μM^−1^ s^−1^ and 0.0403 μM^−1^ s^−1^, respectively. Differential scanning fluorimetry showed that these constructs had similar *in vitro* thermal stabilities (Fig. 3*D*), and limited proteolysis with chymotrypsin suggested that the presence of exon 14 did not significantly alter the protease susceptibility of the structured domains (Fig. 3*E*). Thus, exon 14 inclusion or exclusion, or S481 phosphomutants, do not impact the inherent biochemical properties of ACLY.

**Figure 4:**
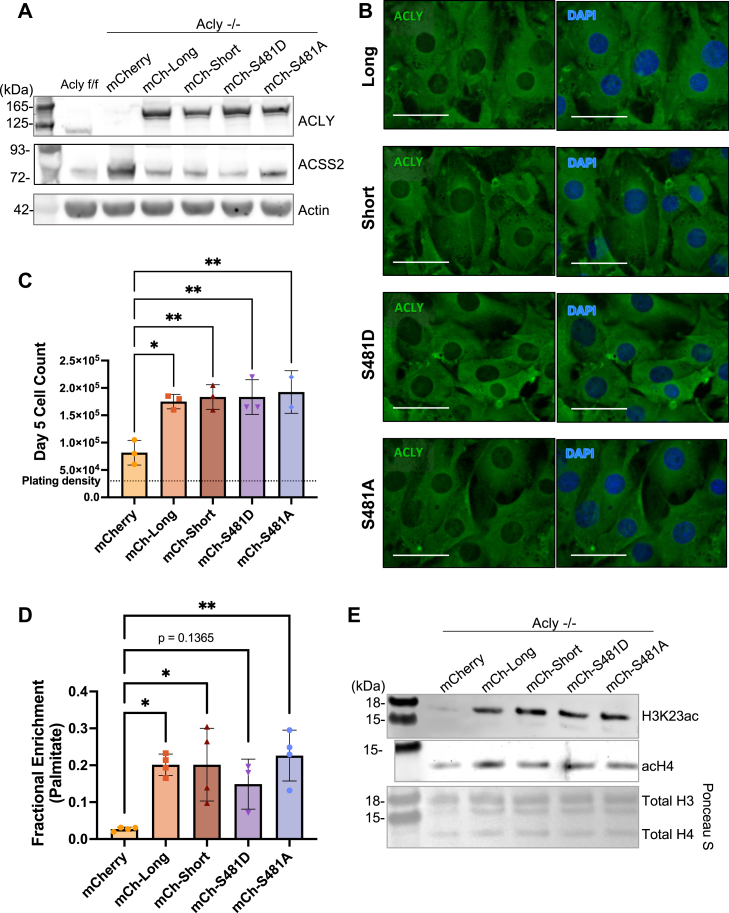
**ACLY isoforms and phosphomutants rescue function in *Acly* knockout cells.***A*, Western blot of mouse embryonic fibroblasts (MEFs) with *Acly* KO and mCherry-tagged human ACLY re-expression. *B*, fluorescence imaging of MEFs expressing indicated ACLY constructs at 60X magnification. Scale bar = 100 μm. *C*, growth of Acly KO MEF cell lines expressing indicated constructs after 5 days in complete media. Data points represent individual plates. Groups were compared using one-way ANOVA. Differences between ACLY addback cell lines were not statistically significant (*p* > 0.05). *D*, ^13^C enrichment in palmitate after 48 h incubation with uniformly labeled ^13^C glucose. Each point represents a biological replicate. Groups were compared using one-way ANOVA. Differences between ACLY addback cell lines were not statistically significant (*p* > 0.05). *E*, Western blot and ponceau stain (*bottom*) of acid-extracted histones. ns (*p* > 0.05), *p* ≤ 0.05 (∗), *p* ≤ 0.01 (∗∗). ACLY, ATP-citrate lyase; KO, knockout.

We considered the possibility that ACLY splicing might impact functions that are not captured *in vitro*, such as protein–protein interactions, employing a cell-based system to evaluate if exon 14 and/or S481 phosphorylation affect known cellular functions of *ACLY*. To this end, we re-expressed mCherry-tagged human ACLY long and short isoforms, and S481D and S481A mutants, in *Acly*-null mouse embryonic fibroblasts, which we previously characterized (39) (Fig. 4*A*). Upon genetic deletion of *Acly*, these cells upregulated ACSS2, which can generate acetyl-CoA directly from acetate and facilitates metabolic compensation. Re-expression of both isoforms and phosphomutants similarly suppresses ACSS2 (Fig. 4*A*). Each protein exhibited similar localization within cells, with primarily cytosolic but also detectable nuclear localization (Fig. 4*B*). Long, short, S481D, and S481A ACLY similarly rescued cell proliferation (Fig. 4*C*), *de novo* palmitate synthesis from uniformly labeled ^13^C-glucose (Fig. 4*D*), and bulk histone acetylation (Fig. 4*E*), indicating that the ACLY isoforms or S481 phosphomutants function comparably to produce acetyl-CoA in cells. ACLY uses citrate derived from the mitochondria, and in some models, such as embryonic stem cells, ACLY expression can determine citrate oxidation in the canonical TCA cycle *versus* export in the citrate–malate shuttle. This shift in citrate utilization can be monitored by the ratio of malate m + 2 to citrate m + 2 labeling from uniformly labeled ^13^C glucose (40). We therefore addressed the potential impact of ACLY splicing on TCA metabolism. However, no significant differences were observed in TCA cycle labeling in mouse embryonic fibroblasts expressing short- or long-isoform ACLY, or ACLY S481A, or S481D mutants, compared to ACLY-deficient mCherry controls (Fig. S3*A*).

Since differences in ACLY splicing patterns is observed in tumors *versus* normal tissues (Fig. 2), we re-expressed each isoform in an *Acly* KO mouse hepatocellular carcinoma cell line (41) to better investigate cancer-specific phenotypes (Fig. S3*B*). Both isoforms rescued cell proliferation (Fig. S3*C*) and histone acetylation in these lines (Fig. S3*B*). The ratio of malate m + 2 to citrate m + 2 labeling from uniformly labeled ^13^C glucose was again unaffected by ACLY isoform re-expression (Fig. S3*D*). Additionally, since tumors *in vivo* are often hypoxic (42, 43), we also measured TCA metabolites under both normoxia and hypoxia (1% O_2_) in the hepatocellular carcinoma cells. Abundance of TCA cycle intermediates, including citrate and malate, was suppressed under hypoxia. Re-expression of either the long or short ACLY isoform lowered levels of ACLY’s substrate citrate in both hypoxia and normoxia (Fig. S3*D*). Minimal changes in malate and α-ketoglutarate were observed with ACLY expression. In summary, the effects of ACLY activity on TCA cycle metabolism appear agnostic to ACLY splicing (Fig. S3*D*).

### *Acly* exon 14 deletion in C57BL6/J mice minimally affects metabolic physiology

**Figure 5:**
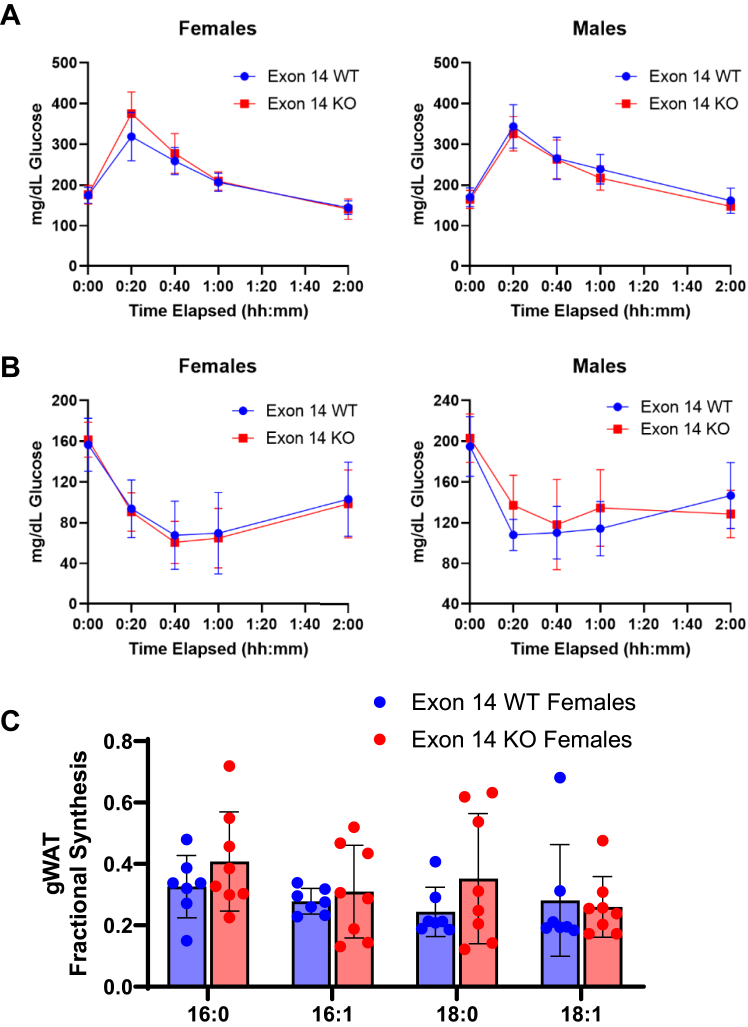
***Acly* exon 14 deletion in C57BL/6J mice minimally affects metabolic physiology.***A*, glucose tolerance test on 6- to 8-week-old mice, n = 8 per group. *B*, insulin tolerance test on 7- to 9-week-old mice, n = 8 per group. *C*, fractional synthesis of fatty acids in gonadal white adipose tissue of female mice after 4 weeks on ZFD (11–13 weeks old). D_2_O was administered for 48 h, and fractional synthesis was calculated. Differences between WT and KO mice were not statistically significant (*p* > 0.05) by two-tailed *t* test. gWAT, gonadal white adipose tissue; KO, knockout; ZFD, zero-fat, high sucrose diet.

To address potential systemic function(s) of *ACLY* splicing, we generated *Acly* exon 14 KO mice on a C57BL/6 background (Fig. S4*A*). ACLY is an essential metabolic enzyme, and germline deletion of *Acly* is embryonic lethal (44). In contrast, *Acly* exon 14 KO mice were born in expected Mendelian ratios and appeared grossly normal. Glucose tolerance test (GTT) and insulin tolerance test (ITT) (Figs. 5, *A* and *B* and S4*B*) revealed no differences between exon 14 KO and WT mice. ACLY is upregulated in fat and liver response to carbohydrate feeding and suppressed by high fat (45, 46), and some *Acly*-dependent phenotypes in mice are not apparent unless the mice are challenged by high levels of carbohydrates (45, 46, 47). We therefore challenged exon 14 KO and WT mice with a zero-fat, high sucrose diet (ZFD) for 4 weeks. We previously reported that adipocyte-specific *Acly* KO mice lose fat mass and develop insulin resistance on ZFD (46), but no differences from WT were observed in exon 14 KO mice in body weight (Fig. S4*C*), mass of gonadal or subcutaneous (inguinal) white adipose tissue depots, or liver mass (Fig. S4*D*). In our previous work, mice on ZFD lacking *Acly* in adipose tissue had reduced *de novo* synthesized fatty acids in gonadal white adipose tissue compared to controls, especially in females (46). However, no differences were observed in fatty acid fractional synthesis between female exon 14 KO mice on ZFD and their WT counterparts (Fig. 5*C*). Overall, the data indicate that exon 14 is not required for ACLY’s major functions in metabolic physiology.

### Acly exon 14 deletion does not impact tumor growth in ApcMin/+ mice

To test if *Acly* exon 14 directly supports tumor growth or cancer phenotypes, we cross-bred our *Acly* exon 14 KO mice to the well-established ApcMin/+ genetic model of intestinal neoplasia. We chose an intestinal tumor model because colon tumors in TCGA exhibit a relatively high *ACLY* PSI along with striking differences in PSI between tumor *versus* normal colon tissue (Fig. 2*A*, colon adenocarcinoma, and Fig. 2*E*). Additionally, several metabolomic and lipidomic analyses of human colorectal cancers have shown that these tumors have altered acyl-CoA and lipid metabolism, which may make them sensitive to ACLY perturbations (48, 49, 50).

**Figure 6:**
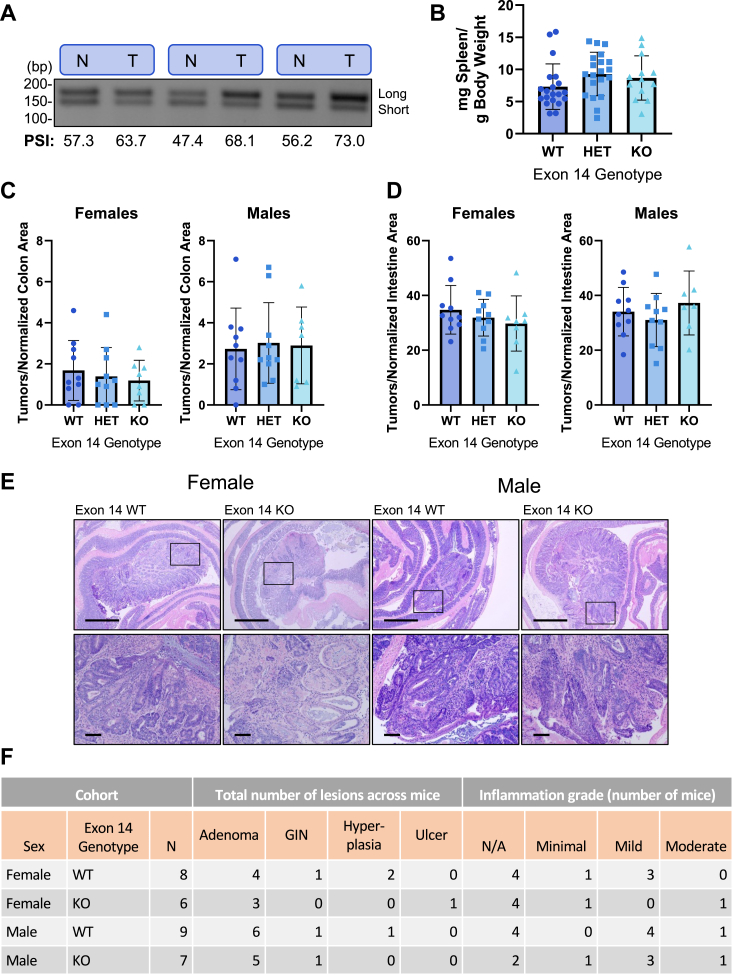
***Acly* exon 14 deletion does not impact tumor growth in ApcMin/+ mice.***A*, RT-PCR on colon tumors (T) and adjacent normal tissue (N) from 13-week-old ApcMin/+ mice. *B*, spleen weight normalized to body weight of tumor-bearing mice. Number of tumors normalized to (*C*) colon and (*D*) small intestinal area. Differences between genotypes were not statistically significant (*p* > 0.05) by one-way ANOVA. *E*, swiss-rolled and H&E-stained colons from representative tumor-bearing mice; *F*, summary of histological findings in tumor-bearing, H&E-stained colons evaluated by veterinary pathologist. Top: 4X magnification, scale bar = 1 mm. Bottom: 20X magnification of 4X area indicated by square, scale bar = 100 μm. ACLY, ATP-citrate lyase; PSI, percent spliced in.

Consistent with TCGA data, tumors from ApcMin/+ mice showed higher PSI than surrounding normal tissue (Fig. 6*A*). However, exon 14 deletion did not impact tumor burden in the colon (Fig. 6*C*) or small intestine (Fig. 6*D*) in either sex. Spleen weight, which can also be used as a proxy for tumor burden (51, 52), was also unchanged with exon 14 deletion (Fig. 6*B*). Colons from ApcMin/+ exon 14 KO and WT mice were Swiss-rolled, fixed, and H&E stained for histology (Fig. 6*E*). A blinded analysis by a veterinary pathologist did not reveal notable differences in tumor histology or inflammation between genotypes (Fig. 6*F*). Thus, the absence of *ACLY* exon 14 alone is not sufficient to alter tumor phenotypes.

### ACLY is regulated by the splicing factor ESRP1

**Figure 7:**
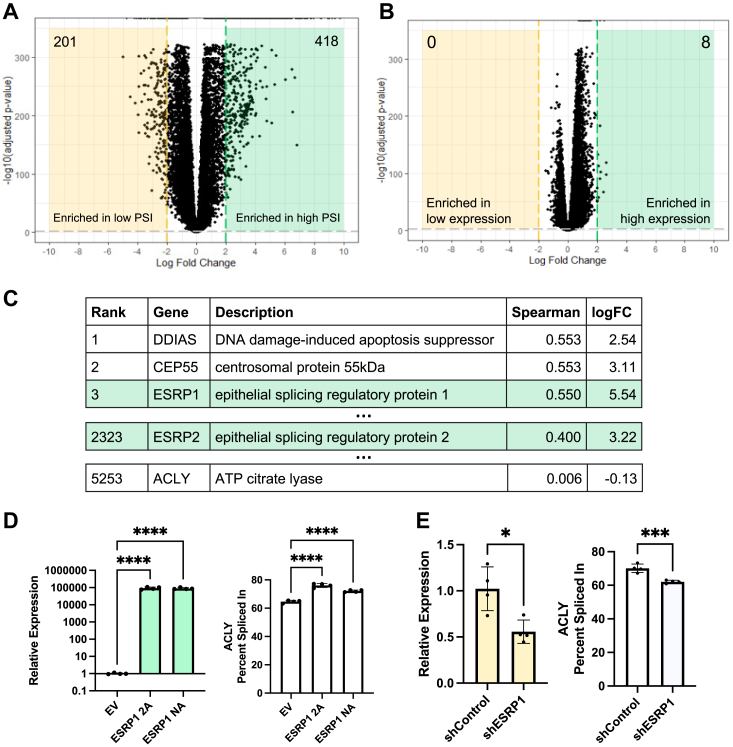
***ACLY* is a target of the splicing factor ESRP1.***A* and *B*, volcano plot of differentially expressed genes in high *versus* low ACLY PSI (*A*) or high *versus* low ACLY expression (*B*). Tumors were stratified by upper and lower quartile (See Fig. 2, *C* and *D*). *C*, genes ranked by Spearman correlation between expression and *ACLY* PSI across TCGA tumor specimens. Log_2_ fold change for selected genes are also included (see (*A*)). *D*, qPCR of ESRP1 normalized by 18S (*left*) and ACLY PSI (*right*) in HEK293T cells overexpressing ESRP1. Data points represent individual wells. Conditions were compared using one-way ANOVA. *E*, qPCR of ESRP1 normalized by 18S (*left*) and ACLY PSI (*right*) in HCT-116 cells expressing indicated shRNA. Data points represent individual wells. Conditions were compared using Student’s two-tailed *t* test. Symbols: ns (*p* > 0.05), *p* ≤ 0.05 (∗), *p* ≤ 0.001 (∗∗∗), *p* ≤ 0.0001 (∗∗∗∗). ACLY, ATP-citrate lyase; ESRP1, epithelial splicing regulatory protein 1; EV, empty vector; PSI, percent spliced in; TCGA, The Cancer Genome Atlas.

With limited evidence that *ACLY* splicing impacts its canonical functions, we returned to TCGA to generate a new hypothesis to explain the association between *ACLY* PSI, cancer, and patient survival (Fig. 2). Overall, tumors stratified by *ACLY* PSI had over 600 differentially expressed genes (Fig. 7*A*), while the tumors stratified by total *ACLY* expression only had eight differentially expressed genes (Fig. 7*B*). By analyzing the correlation between *ACLY* PSI and each detected transcript, we found that *ESRP1* has the third highest Spearman correlation and is highly expressed in high PSI *versus* low PSI tumors (Fig. 7*C*). ESRP1 is a splicing factor that helps establish epithelial cell identity and is implicated in epithelial–mesenchymal transition in tumors (53). *ESRP1* itself has multiple splice isoforms; overexpression of either major isoform (known as 2A and NA) in HEK293T cells (Fig. 7*D*, left) resulted in increased *ACLY* PSI (Figs. 7*D*, right and S4*A*, top). As a control, we also examined the canonical ESRP1 target *FGFR2* (53), which showed an isoform switch upon 2A or NA overexpression as expected (Fig. S5*A*, bottom). Alternatively, shRNA knockdown of *ESRP1* in HCT-116 cells (Fig. 7*E*, left) resulted in lower *ACLY* PSI (Figs. 7*E*, right and S5*B*, left) and exon skipping in the canonical target *CD44* (53) (Fig. S5*B*, right). These data indicate that ESRP1 is one factor that influences *ACLY* alternative splicing. Interestingly, *ESRP1* expression is not correlated with poor survival in a pan-cancer analysis (Fig. S5*C*), in contrast to *ACLY* PSI, suggesting that additional factors may influence *ACLY* splicing and associated phenotype(s).

To identify potential functional consequences of ESRP1-directed *ACLY* splicing, we performed gene set enrichment analysis comparing high *versus* low *ACLY* PSI tumors (54). The most enriched gene sets corresponded to signatures characteristic of an immunosuppressive microenvironment, particularly IL-10 signaling and tumor-associated macrophages, mast cells, and B cells, as well as cell cycle–related gene sets (Tables S1 and S2). Re-analysis comparing high *versus* low *ESRP1*-expressing tumors showed very similar enrichment patterns (Tables S1 and S2). Thus, *ACLY* splicing may be component of an *ESRP1*-dependent program that relates cancer phenotypes.

## Discussion

In this study, we report the expression of long and short ACLY splice isoforms in mammalian tissues, along with evidence that exon 14 inclusion is increased in multiple cancer types. To our knowledge, this is the first study to specifically characterize the short isoform of ACLY. Despite these intriguing expression patterns, we found that exon 14 does not appear to be essential to any of ACLY’s canonical functions *in vitro*, in cells or in mice. However, several metabolic enzymes are reported to have “moonlighting” functions (55), and it remains possible that *ACLY* splicing is relevant only in specific physiological contexts which we did not evaluate. An illustrative example may again be alternative splicing of pyruvate kinase (specifically the PKM2 isoform) which enhances tumor growth in some cancer models as discussed earlier but is dispensable in others (56, 57, 58). In addition, our *in vitro* assays were carried out using recombinant protein purified from *E. coli* with an amino acid substitution (aspartate) at serine 481 to simulate phosphorylation. Aspartate may not adequately mimic the phosphorylated state in this context, or we may be missing other relevant post-translational modifications such as acetylation or other phosphorylation sites.

A major motivation for this study was our initial survey of patient data, which suggested that high *ACLY* PSI is prevalent in cancer and is associated with deadlier tumors overall. Strikingly, most samples in each tumor type can be binned into a single PSI category, in contrast to total *ACLY*. These data suggest that PSI is regulated at the tissue level and reinforces the idea that *ACLY* expression and *ACLY* splicing are distinct molecular events that may have divergent functional roles. We hoped that a functional analysis of these isoforms would help explain why tumor types with high *ACLY* PSI tend to be deadlier regardless of tissue origin, stage, or treatment modality. Even though the intestinal tumors in our mouse model had higher ACLY PSI than adjacent normal tissue, much like their human counterparts, ACLY exon 14 KO did not attenuate tumor formation or growth. It could be that ACLY splicing by itself is not sufficient to affect tumor growth, but rather, that ACLY splicing is one component of a larger biological program that our correlational analysis was unable to fully capture.

We provide evidence that *ACLY* splicing is regulated by ESRP1. ESRP1 manipulation does not result in complete *ACLY* isoform “switching,” which is expected considering that most tissues express both isoforms to some degree (Figs. 1 and S1). Other splicing factors such as ESRP2, which has some functions redundant to ESRP1 (53), may also regulate *ACLY* alternative splicing. Notably, *ESRP1* germline KO in mice results in a severe cleft palate phenotype leading to neonatal death (59). Since *Acly* exon 14 KO mice did not have this phenotype, ESRP1 is likely modulating *ACLY* splicing and not *vice versa*.

Specimens in TCGA are bulk tumor which include tumor cells as well as immune cells, cancer-associated fibroblasts, and other components of the tumor microenvironment. It is possible that an increase in *ACLY* PSI in bulk tumor could be a consequence of immune infiltration. However, homogeneous human cancer cell lines tend to have quite high *ACLY* PSI (Fig. 1*B*), and ESRP1 and 2 are exclusively expressed in epithelial cells (53). Although ESRP1 in cancer is primarily documented to be involved in EMT and metastasis (60, 61), an *in silico* analysis found ESRP1 to be a target of interest in overcoming immune evasion in melanoma (62). Another study on ESRP1 in ovarian cancer showed that *ESRP1* overexpression in tumor cells resulted in an immunosuppressive microenvironment (63). Notably, the immune signatures that we find closely correlated with *ESRP1* expression and *ACLY* PSI are typically protumor and oppose cytotoxic T cell responses, especially through the immunosuppressive cytokine IL-10 (64, 65). *ESRP1* expression has been reported to have both protumor and antitumor roles in colorectal cancer *in vivo* (66, 67), but these studies were done in immunocompromised mouse models. ESRP1 has multiple validated (and even more suspected) splicing targets which may connect ESRP1 expression and immune infiltration. *ACLY* exon 14 inclusion may be providing a functional redundancy in such a mechanism or be concurrent but functionally independent. The connection between ESRP1 and immunosuppression warrants further investigation but is outside the scope of this study.

The structure of intact ACLY from various species has been determined by both X-ray crystallography (68) and single-particle analysis cryo-electron microscopy (13, 24). While cryo-electron microscopy studies have succeeded using full-length protein, crystallography studies removed the IDR, including exon 14, to aid in crystallization. These structures are quite similar, and ACLY that is missing the IDR has a similar catalytic rate to the full-length protein (68) which supports our findings here that modifications to exon 14 do not impact catalytic activity. Intriguingly, the IDR in ACLY is only present in the animal kingdom, as most organisms express *ACLY* as two separate transcripts (68, 69). As described above, evolution has taken advantage of phosphorylation sites (S455, T477 & S451) in the IDR to regulate complex biological responses. While the two ACLY splice isoforms are expressed broadly across tissues, are regulated in tumors and associated with cancer phenotypes, and include sites that may be modified post-translationally, the functions of exon 14 remain elusive. We hope that this study sets the stage for a more complete understanding of *ACLY* splicing and the role of *ESRP1* in cancer.

## Experimental procedures

### RNA extraction and reverse-transcription PCR

RNA was extracted from cultured cells and murine tissues using TRIzol reagent (Invitrogen, 15596018) according to the manufacturer’s protocol. One microgram pure RNA (quantified by Nanodrop) was used for cDNA synthesis with the High-Capacity cDNA Reverse Transcription Kit (Applied Biosystems, 4368814). Two microliter undiluted cDNA was used for PCR with GoTaq Master Mix (Promega, M7123). PCR thermocycling conditions were as follows: 95 °C for 5 min; 25 cycles of 95 °C for 30 s, 57 °C for 30 s, 72 °C for 1 min; and hold at 10 °C. PCR products were run on a 1.8% agarose gel in tris-borate-EDTA buffer with 1:10,000 EtBr at 80 V for 1 h. Imaged bands were quantified by densitometry using ImageJ.

### Expression and purification of ACLY

Codon-optimized human *ACLY* with a C-terminal 6xHis tag in a pET24a vector (24) was expressed in BL21(DE3) cells. Additional constructs were generated using site-directed mutagenesis and confirmed by Sanger sequencing. Transformed *E. coli* were grown under 0.05 mg/ml kanamycin selection at 37 °C until *A* was approximately 0.8. Cultures were induced with 0.1 mg/ml IPTG overnight at 16 °C. Bacteria were pelleted by centrifugation at 4000*g* for 15 min and resuspended in lysis buffer (25 mM Tris-HCl, pH 7.5, 500 mM NaCl, and 10% glycerol). Pellets were flash frozen in liquid nitrogen and stored at −80 °C until purification. Thawed pellets in lysis buffer were sonicated at 70% amplitude for 2 min (10 s on and 20 s off), and lysate was clarified by centrifugation at 19,000 rpm for 30 min. After equilibration with lysis buffer, lysate was passed through 2 ml TALON Metal Affinity Resin (Takara, 635504). The column was then washed with 20 ml lysis buffer and 20 ml lysis buffer with 40 mM imidazole. Protein was eluted with lysis buffer with 200 mM imidazole and further purified by gel filtration using a Superdex 200 10/30 Gl column (GE healthcare) in 25 mM Hepes pH 7.5, 200 mM NaCl, 10 mM βME, and 1 mM MgCl_2_. Aliquots were flash frozen with 5 to 10% glycerol in liquid nitrogen and stored at −80 °C. Before use, protein was dialyzed for at least 2 h in 25 mM Hepes, pH 7.5, 200 mM NaCl, and 10 mM βME at 4 °C.

### ACLY-coupled enzymatic activity assay

Activity of 50 nM *ACLY* was determined by coupling to malate dehydrogenase, which reduces oxaloacetate to malate using NADH. The reaction buffer consisted of 100 mM Hepes pH 8.0, 200 mM NaCl, 1 mM DTT, 1 mM ATP, 1 mM MgCl_2_, 1 mM sodium citrate, 0.2 mM CoA, 0.2 mM NADH, 10 U MDH (Millipore, 442610), and 0.01% NP-40. The reaction was initiated by the final addition of CoA, and then, absorbance at 340 nm was immediately monitored every 30 s for 10 min to measure the consumption of NADH.

### Differential scanning fluorimetry

Purified *ACLY* (5 μM) in 25 mM Hepes, pH 7.5, 200 mM NaCl, and 10 mM βME was mixed with 5X SYPRO Orange (Invitrogen, S6650) to a final volume of 20 μL. The fluorescence of triplicate mixes along with negative controls (without protein and/or without SYRRO orange) were measured from 25 °C to 90 °C using a qPCR instrument at a 1 °C/min ramp rate. Data were analyzed and graphed using DSF World (70) with sigmoid fitting (Fit 1).

### Limited proteolysis

Chymotrypsin (Sigma, C3142) was prepared by dilution into 1 mM HCl and 2 mM CaCl2. *ACLY* (0.2 mg/ml) was reacted with a 50:1 M equivalent of chymotrypsin in 50 mM Tris HCl, pH 7.5. One microgram *ACLY* aliquots were removed at indicated time points and quenched using SDS-PAGE loading buffer. Samples were boiled for 5 min before SDS-PAGE and subsequent staining with Coomassie blue.

### Cell culture and cell lines

All cell culture was carried out in Dulbecco's modified Eagle's medium (DMEM), high glucose (Gibco, 11965092), and 10% calf serum (Gemini, #100-510) unless otherwise indicated. Details on *Acly* KO mouse embryonic fibroblasts generation are described by Zhao *et al.* (39). Plasmids were constructed by subcloning into the vector backbones described by Raymond *et al.* (71) and confirmed by whole-plasmid sequencing. Stable re-expression of human *ACLY* tagged with mCherry was achieved by lentiviral transduction, and mCherry positive cells were selected by fluorescence-activated cell sorting. Detailed plasmid maps confirmed by whole-plasmid sequencing can be found in the supplementary methods. Transient transfection of ESRP1 expression plasmids (see Warzecha *et al.* (53)) was achieved using Lipofectamine 2000 (Invitrogen, 11668019) in HEK293T cells according to the manufacturers protocol. shRNA in pGIPZ (Horizon Discovery) was expressed in HCT-116 cells by lentiviral transduction. Forty-eight hours after transduction, plasmid-containing cells were selected with 1 ug/ml puromycin for 48 h. ESRP1 expression and knockdown plasmids were a generous gift from Natoya Peart (Department of Biology, University of Waterloo).

### Protein extraction

Cell pellets were resuspended in lysis buffer (20 mM Tris-HCl pH 7.9, 20% glycerol, 420 mM NaCl, 1.5 mM MgCl_2_, 0.1% NP-40, 0.2 mM EDTA, 0.5 mM DTT, 0.2 mM PMSF, and 1X protease inhibitor cocktail) and incubated for 20 min at 4 °C on a rotating platform. An equivalent volume of low-salt buffer (10 mM Hepes pH 7.5, 1.5 mM MgCl2, 10 mM KCl, and 1X protease inhibitor cocktail) was added. The lysate was clarified by centrifugation at 14,000 rpm for 10 min at 4 °C. Lysates were stored at −80 °C until analysis.

### Immunofluorescence

Cells were cultured on glass-bottom TC plates for imaging. Once adhered, cells were washed three times with PBS and fixed in 4% paraformaldehyde for 15 min. After fixation, cells were washed three times in tris-buffered saline with 0.1% tween 20 and blocked for 1 h (10% goat serum, 1% bovine serum albumin, 0.1% gelatin, 22.52 mg/ml glycine, 0.5% Triton X in TBST). Cells were then incubated with 1:1000 *ACLY* antibody (Abcam, ab40793) in 5% animal serum and 0.1% Triton X in TBST for at least 3 h. After washing three times with TBST, cells were incubated with Alexa488-conjugated secondary antibody diluted 1:500 and DAPI diluted 1:500 in 5% animal serum and 0.1% Triton X in TBST for 1 h. Finally, cells were washed with TBST three times before imaging on an EVOS m5000 imaging system.

### *De novo* lipogenesis in cultured cells

For ^13^C-glucose tracing, 500,000 cells were plated in complete media. After 24 h, media were changed to DMEM containing no glucose, glutamine, pyruvate, and phenol red (Gibco, A1443001) with 10% dialyzed FBS (Gemini, 100-108) supplemented with 10 mM [U-^13^C]-glucose and 4 mM glutamine. Cells were collected after 48 h by trypsinization and neutralization in PBS with 1% fatty acid–free BSA. Cells were pelleted and washed with PBS prior to storage at −80 °C.

To extract lipids, 2 ml methanol, 1.7 ml chloroform, and 1.4 ml glass-distilled water were added to cell pellets. Ten microliter of 1 mM heptadecanoic acid was added as an internal standard. The mixture was vortexed and centrifuged for 5 min at 2000 rpm. The chloroform fraction was moved to a new tube, and the aqueous fraction was re-extracted by addition of 0.7 ml chloroform followed by vortexing and centrifugation. Combined chloroform fractions were back extracted by addition of 0.1 ml water followed by vortexing and centrifugation. Chloroform-dissolved lipids were dried under a nitrogen stream. Lipids were derivatized into methyl esters and measured by GC/MS using an Agilent GC-MS 7890A/5975A with a DB5 column as previously described (41). Isotope enrichment was calculated using IsoCor v2 (72).

### Acid extraction of histones

Cells were scraped into 1 ml cold hypotonic lysis buffer (10 mM Tris-HCl pH 8.0, 1 mM KCl, 1.5 mM MgCl_2_, 0.1% Triton X, 1X protease inhibitor cocktail) and incubated on a rotating platform for 30 min at 4 °C. Nuclei were then pelleted by centrifugation at 6500*g* for 10 min at 4 °C, washed with 0.5 ml hypotonic lysis buffer, and resuspended in 0.2 M HCl. The resuspension was incubated overnight on a rotating platform at 4 °C. Nuclear debris was pelleted by centrifugation at 6500*g* for 10 min at 4 °C. Histones (supernatant) were neutralized by addition of 1:10 volume of 2M NaCl and stored at −80 °C until analysis.

### Analysis of TCA metabolites

For ^13^C glucose tracing, cells were plated in complete media. The following day, cells and tracing media were either equilibrated at 1% O_2_ in a Whitley H35 hypoxystation or kept in normal atmosphere for 24 h. Then, media were changed to DMEM containing no glucose, glutamine, pyruvate, and phenol red (Gibco, A1443001) with 10% dialyzed FBS (Gemini, 100-108) supplemented with 10 mM [U-^13^C]-glucose and 4 mM glutamine. After 6 h, cells were washed twice with cold PBS, scraped into ice cold 80% methanol, and flash frozen in liquid nitrogen. After 1 μM norvaline spike-in, cells went through three cycles of freeze-thaw in liquid nitrogen with periodic shaking. Cell debris was centrifuged out at max speed for 15 min, and supernatant containing polar metabolites was dried down overnight in a SpeedVac.

For trimethylsilyl derivatization, dried metabolites were resuspended in 30 μL methoxyamine in pyridine (5 mg/ml), vortexed, and incubated at 70 °C for 15 min. Then, 70 μL N-tert-butyldimethylsilyl-N-methyltrifluoroacetamide (Thermo Scientific, TS-48920) was added. The samples were vortexed and incubated at 70 °C for 1 h. After derivatization, samples were centrifuged at max speed for 10 min and the top 50 μL transferred to glass vials for analysis by GC-MS on an Agilent GC-MS 7890A/5975A with a DB-5 column. Isotope natural abundance correction was performed with FluxFix using an unlabeled control (73). Relative quantification was performed by normalizing the area under the curve of each species to the internal standard and subsequently the total ion counts of the same sample.

### Generation of Acly exon 14 null mice

Mouse work was performed in accordance with Institutional Animal Care and Use Committee guidelines under an approved protocol. Deletion of *Acly* exon 14 in C57BL6/J mice was performed by the CRISPR/Cas9 Mouse Targeting Core at the University of Pennsylvania. Founder mice were screened by PCR, and the presence of the exon 14 KO allele confirmed by Sanger sequencing.

### Glucose and insulin tolerance test

GTTs and ITTs were performed on mice at 6 to 8 weeks of age. Mice were weighed, individually housed, and fasted for 4 h prior to the experiment. Dextrose (2.0 g/kg) or 0.5 U/kg insulin was IP injected for GTT or ITT, respectively. Blood glucose was measured by tail vein bleed using a glucometer at indicated time points. For GTT, additional blood was collected at 0 and 20 min relative to injection to measure insulin response. Blood was centrifuged at 2600*g* for 15 min, and plasma was stored at −80 °C until insulin could be measured by ELISA (Crystal Chem, 90080) according to the manufacturer’s instructions.

### *In vivo de novo* lipogenesis

Zero-fat diet (62% sucrose) chow was obtained from Envigo (TD.03314). Mice were euthanized at 10 to 12 weeks of age using a carbon dioxide chamber for tissue collection. For deuterium labeling in mouse tissues, mice were given 20% deuterium in drinking water (^2^H_2_O) for 48 h prior to euthanasia. Tissues were immediately flash frozen in liquid nitrogen upon euthanasia and dissection. Ten milligram tissue was lipid-extracted, derivatized, and analyzed by GC/MS (as described *in vitro*). Deuterium enrichment in fatty acids was normalized to plasma D_2_O enrichment levels as follows: blood plasma was collected at sac by cardiac puncture and centrifugation for 15 min at 2600*g*, and deuterium content was determined by acetate exchanged as previously described (39).

### ApcMin/+ mouse tumor experiments

ApcMin/+ males in a C57BL6/J background (Jackson Laboratory, #002020), which spontaneously form intestinal tumors with 100% penetrance, were bred to Apc WT females. Tumor-bearing mice were regularly monitored for complications. Experimental mice were euthanized at 13 weeks of age. Nonexperimental mice were euthanized before 100 days of age to prevent excessive tumor burden. Euthanasia was performed using a carbon dioxide chamber.

### Intestinal tumor quantification and histology

Mouse colon or the distal third of the small intestine was first flushed with PBS using a gavage needle to remove fecal matter and then flushed with Boulin’s fixative (50% ethanol and 5% acetic acid in water). Intestines were cut transverse, laid flat with luminal side up, and photographed using an iPhone 14 with a ruler to standardize frame size. Later, relative tissue area was measured, and tumors were counted using ImageJ. Tumor count was then normalized to visible tissue area. The intestine was Swiss rolled, placed into histology cassettes, and fixed in 4% paraformaldehyde for 24 h. Tissues were dehydrated in 50% and then 70% ethanol for at least 1 day. Tissues were then stored in 70% ethanol until paraffin embedding, sectioning, and H&E staining by the Molecular Pathology and Imaging Core at the University of Pennsylvania. Blinded H&E sections were evaluated by the Comparative Pathology Core at the University of Pennsylvania School of Veterinary Medicine.

### Real-time qPCR

cDNA was diluted in nuclease-free water 1:20 for all genes except the 18S reference gene, which was diluted 1:500. Diluted cDNA (2 μL) was added to quadruplicate reactions containing 1X Power SYBR Green PCR Master Mix (Applied Biosystems, 4367659) and 0.5 μM each primer up to 20 μL final volume. No DNA controls were included on each run. Thermocycling and fluorescence detection was performed using a Viia 7 Real-Time PCR System with the standard 2-step protocol for 40 cycles.

### Primer sequences

**Table:** 

Target	Purpose	Forward sequence	Reverse sequence
Human *ACLY* isoforms	RT-PCR	AAGCCTGCCATGCCACAAG	AGCCACTGAGGGCTCGTCTC
Mouse *ACLY* isoforms	RT-PCR	GAGTCCCGAGCTGATGAAGT	AGCCACTGAGGGCTCGTCTC
Human FGFR2 isoforms (53)	RT-PCR	TGGATCAAGCACGTGGAAAAGA	GGCGATTAAGAAGACCCCTATGC
Human CD44 isoforms (53)	RT-PCR	AGGAGCAGCACTTCAGGAGGTTAC	ACTGGGGTGGAATGTGTCTTGGTC
Human ESRP1 (Origene)	qPCR	CTCAGGGTCGAAGGAACGGA	TACCGGGTCCCCATGTGATG

### Antibodies

**Table:** 

Target	Purpose	Manufacturer	Cat. No.
*ACLY*	Western blot	Proteintech	15421-1-AP
*ACLY*	Immunofluorescence	Abcam	ab40793
H3K23ac	Western blot	Cell Signaling Technologies	14932
H4ac	Western blot	Millipore	06-866
Actin	Western blot	Cell Signaling Technologies	3700S

## Data availability

The data generated in this study are available upon request from the corresponding authors. Expression profile data analyzed in this study were obtained from The Cancer Genome Atlas, and the code used to analyze this data is available in the supplementary data files.

## Supporting information

This article contains supporting information.(38, 53)

## Conflict of interest

The authors declare that they have no conflicts of interest with the contents of this article.

## References

[1] Liberti M.V., Locasale J.W. (2016). The Warburg effect: how does it benefit cancer cells?. Trends Biochem. Sci..

[2] Oltean S., Bates D.O. (2014). Hallmarks of alternative splicing in cancer. Oncogene.

[3] Elgadi K.M., Meguid R.A., Qian M., Souba W.W., Abcouwer S.F. (1999). Cloning and analysis of unique human glutaminase isoforms generated by tissue-specific alternative splicing. Physiol. Genomics.

[4] Li X., Qian X., Peng L.-X., Jiang Y., Hawke D.H., Zheng Y. (2016). A splicing switch from ketohexokinase-C to ketohexokinase-A drives hepatocellular carcinoma formation. Nat. Cell Biol..

[5] Christofk H.R., Vander Heiden M.G., Harris M.H., Ramanathan A., Gerszten R.E., Wei R. (2008). The M2 splice isoform of pyruvate kinase is important for cancer metabolism and tumour growth. Nature.

[6] Anastasiou D., Poulogiannis G., Asara J.M., Boxer M.B., Jiang J., Shen M. (2011). Inhibition of pyruvate kinase M2 by reactive oxygen species contributes to cellular Antioxidant responses. Science.

[7] Yang W., Xia Y., Ji H., Zheng Y., Liang J., Huang W. (2011). Nuclear PKM2 regulates β-catenin transactivation upon EGFR activation. Nature.

[8] Luo W., Hu H., Chang R., Zhong J., Knabel M., O’Meally R. (2011). Pyruvate kinase M2 is a PHD3-stimulated coactivator for hypoxia-Inducible factor 1. Cell.

[9] Anastasiou D., Yu Y., Israelsen W.J., Jiang J., Boxer M.B., Hong B.S. (2012). Pyruvate kinase M2 activators promote tetramer formation and suppress tumorigenesis. Nat. Chem. Biol..

[10] Luengo A., Gui D.Y., Vander Heiden M.G. (2017). Targeting metabolism for cancer therapy. Cell Chem. Biol..

[11] Wellen K.E., Hatzivassiliou G., Sachdeva U.M., Bui T.V., Cross J.R., Thompson C.B. (2009). ATP-citrate lyase links cellular metabolism to histone acetylation. Science.

[12] Li J.J., Wang H., Tino J.A., Robl J.A., Herpin T.F., Lawrence R.M. (2007). 2-Hydroxy-N-arylbenzenesulfonamides as ATP-citrate lyase inhibitors. Bioorg. Med. Chem. Lett..

[13] Wei J., Leit S., Kuai J., Therrien E., Rafi S., Harwood H.J. (2019). An allosteric mechanism for potent inhibition of human ATP-citrate lyase. Nature.

[14] Hu J., Komakula A., Fraser M.E. (2017). Binding of hydroxycitrate to human ATP-citrate lyase. Acta Crystallogr. D Struct. Biol..

[15] Sola-García A., Cáliz-Molina M.Á., Espadas I., Petr M., Panadero-Morón C., González-Morán D. (2023). Metabolic reprogramming by Acly inhibition using SB-204990 alters glucoregulation and modulates molecular mechanisms associated with aging. Commun. Biol..

[16] Krishna Mohan G.V., Chenna V.S.H., Tirumandyam G., Mian A.R., Rashid A., Saleem F. (2023). Efficacy and Safety of Bempedoic acid to prevent Cardiovascular events in individuals at Risk of Cardiovascular diseases: a Meta-analysis of Randomized-control Trials. Cureus.

[17] Qian X., Hu J., Zhao J., Chen H. (2015). ATP citrate lyase expression is associated with advanced stage and prognosis in gastric adenocarcinoma. Int. J. Clin. Exp. Med..

[18] Teng L., Chen Y., Cao Y., Wang W., Xu Y., Wang Y. (2018). Overexpression of ATP citrate lyase in renal cell carcinoma tissues and its effect on the human renal carcinoma cells in vitro. Oncol. Lett..

[19] Wen J., Min X., Shen M., Hua Q., Han Y., Zhao L. (2019). ACLY facilitates colon cancer cell metastasis by CTNNB1. J. Exp. Clin. Cancer Res..

[20] Hatzivassiliou G., Zhao F., Bauer D.E., Andreadis C., Shaw A.N., Dhanak D. (2005). ATP citrate lyase inhibition can suppress tumor cell growth. Cancer Cell.

[21] Carrer A., Trefely S., Zhao S., Campbell S.L., Norgard R.J., Schultz K.C. (2019). Acetyl-CoA metabolism supports multistep pancreatic tumorigenesis. Cancer Discov..

[22] Lin R., Tao R., Gao X., Li T., Zhou X., Guan K.-L. (2013). Acetylation Stabilizes ATP-citrate lyase to promote lipid Biosynthesis and tumor growth. Mol. Cell.

[23] Wei X., Shi J., Lin Q., Ma X., Pang Y., Mao H. (2021). Corrigendum: targeting ACLY attenuates tumor growth and Acquired Cisplatin resistance in ovarian cancer by inhibiting the PI3K–AKT pathway and Activating the AMPK–ROS pathway. Front. Oncol..

[24] Wei X., Schultz K., Bazilevsky G.A., Vogt A., Marmorstein R. (2020). Molecular basis for acetyl-CoA production by ATP-citrate lyase. Nat. Struct. Mol. Biol..

[25] Lee J.V., Carrer A., Shah S., Snyder N.W., Wei S., Venneti S. (2014). Akt-dependent metabolic reprogramming regulates tumor cell histone acetylation. Cell Metab..

[26] Sivanand S., Rhoades S., Jiang Q., Lee J.V., Benci J., Zhang J. (2017). Nuclear acetyl-CoA production by ACLY promotes homologous recombination. Mol. Cell.

[27] Martinez Calejman C., Trefely S., Entwisle S.W., Luciano A., Jung S.M., Hsiao W. (2020). mTORC2-AKT signaling to ATP-citrate lyase drives brown adipogenesis and de novo lipogenesis. Nat. Commun..

[28] Ramakrishna S., Pucci D.L., Benjamin W.B. (1983). Dependence of ATP-citrate lyase kinase activity on the phosphorylation of ATP-citrate lyase by cyclic AMP-dependent protein kinase. J. Biol. Chem..

[29] Daub H., Olsen J.V., Bairlein M., Gnad F., Oppermann F.S., Körner R. (2008). Kinase-selective enrichment Enables quantitative phosphoproteomics of the Kinome across the cell cycle. Mol. Cell.

[30] Olsen J.V., Vermeulen M., Santamaria A., Kumar C., Miller M.L., Jensen L.J. (2010). Quantitative phosphoproteomics reveals Widespread full phosphorylation site Occupancy during Mitosis. Sci. Signal..

[31] Dephoure N., Zhou C., Villén J., Beausoleil S.A., Bakalarski C.E., Elledge S.J. (2008). A quantitative atlas of mitotic phosphorylation. Proc. Natl. Acad. Sci. U. S. A..

[32] Yu L.-R., Zhu Z., Chan K.C., Issaq H.J., Dimitrov D.S., Veenstra T.D. (2007). Improved titanium dioxide enrichment of phosphopeptides from hela cells and high confident phosphopeptide identification by cross-validation of MS/MS and MS/MS/MS Spectra. J. Proteome Res..

[33] Zhou H., Di Palma S., Preisinger C., Peng M., Polat A.N., Heck A.J.R. (2013). Toward a Comprehensive characterization of a human cancer cell Phosphoproteome. J. Proteome Res..

[34] Rigbolt K.T.G., Prokhorova T.A., Akimov V., Henningsen J., Johansen P.T., Kratchmarova I. (2011). System-Wide Temporal characterization of the Proteome and Phosphoproteome of human embryonic stem cell differentiation. Sci. Signal..

[35] Olsen J.V., Blagoev B., Gnad F., Macek B., Kumar C., Mortensen P. (2006). Global, in vivo, and site-specific phosphorylation dynamics in signaling networks. Cell.

[36] Mayya V., Lundgren D.H., Hwang S.-I., Rezaul K., Wu L., Eng J.K. (2009). Quantitative phosphoproteomic analysis of T cell Receptor signaling reveals system-wide modulation of protein-protein interactions. Sci. Signal..

[37] Smith L.C., Ralston-Hooper K.J., Ferguson P.L., Sabo-Attwood T. (2016). The G protein-Coupled Estrogen Receptor Agonist G-1 inhibits nuclear Estrogen Receptor activity and Stimulates novel phosphoproteomic signatures. Toxicol. Sci..

[38] Tapial J., Ha K.C.H., Sterne-Weiler T., Gohr A., Braunschweig U., Hermoso-Pulido A. (2017). An atlas of alternative splicing profiles and functional associations reveals new regulatory programs and genes that simultaneously express multiple major isoforms. Genome Res..

[39] Zhao S., Torres A., Henry R.A., Trefely S., Wallace M., Lee J.V. (2016). ATP-citrate lyase controls a glucose-to-acetate metabolic switch. Cell Rep..

[40] Arnold P.K., Jackson B.T., Paras K.I., Brunner J.S., Hart M.L., Newsom O.J. (2022). A non-canonical tricarboxylic acid cycle underlies cellular identity. Nature.

[41] Izzo L.T., Trefely S., Demetriadou C., Drummond J.M., Mizukami T., Kuprasertkul N. (2023). Acetylcarnitine shuttling links mitochondrial metabolism to histone acetylation and lipogenesis. Sci. Adv..

[42] Metallo C.M., Gameiro P.A., Bell E.L., Mattaini K.R., Yang J., Hiller K. (2011). Reductive glutamine metabolism by IDH1 mediates lipogenesis under hypoxia. Nature.

[43] Wise D.R., Ward P.S., Shay J.E.S., Cross J.R., Gruber J.J., Sachdeva U.M. (2011). Hypoxia promotes isocitrate dehydrogenase-dependent carboxylation of α-ketoglutarate to citrate to support cell growth and viability. Proc. Natl. Acad. Sci. U. S. A..

[44] Beigneux A.P., Kosinski C., Gavino B., Horton J.D., Skarnes W.C., Young S.G. (2004). ATP-citrate lyase deficiency in the mouse. J. Biol. Chem..

[45] Carrer A., Parris J.L.D., Trefely S., Henry R.A., Montgomery D.C., Torres A. (2017). Impact of a high-fat diet on tissue acyl-CoA and histone acetylation levels. J. Biol. Chem..

[46] Fernandez S., Viola J.M., Torres A., Wallace M., Trefely S., Zhao S. (2019). Adipocyte ACLY facilitates dietary carbohydrate Handling to maintain metabolic Homeostasis in females. Cell Rep..

[47] Zhao S., Jang C., Liu J., Uehara K., Gilbert M., Izzo L. (2020). Dietary fructose feeds hepatic lipogenesis via microbiota-derived acetate. Nature.

[48] Wang Y., Hinz S., Uckermann O., Hönscheid P., von Schönfels W., Burmeister G. (2020). Shotgun lipidomics-based characterization of the landscape of lipid metabolism in colorectal cancer. Biochim Biophys Acta Mol. Cell Biol. Lipids.

[49] Mika A., Pakiet A., Czumaj A., Kaczynski Z., Liakh I., Kobiela J. (2020). Decreased Triacylglycerol content and elevated contents of cell Membrane lipids in colorectal cancer tissue: a lipidomic study. J. Clin. Med..

[50] Zhu Y., Zhou H., Chen H., Zhang J., Liang Y., Yang S. (2023). Global serum metabolomic and lipidomic analyses reveal lipid perturbations and potential biomarkers of the colorectal cancer by adenoma-carcinoma sequence. Chin. J. Anal. Chem..

[51] Shepherd A.L., Smith A.A.T., Wakelin K.A., Kuhn S., Yang J., Eccles D.A. (2020). A semi-automated technique for adenoma quantification in the ApcMin mouse using FeatureCounter. Sci. Rep..

[52] Hodgson A., Wier E.M., Fu K., Sun X., Wan F. (2016). Ultrasound imaging of splenomegaly as a proxy to monitor colon tumor development in Apc min716/+ mice. Cancer Med..

[53] Warzecha C.C., Sato T.K., Nabet B., Hogenesch J.B., Carstens R.P. (2009). ESRP1 and ESRP2 are epithelial cell-type-specific Regulators of FGFR2 splicing. Mol. Cell.

[54] Subramanian A., Tamayo P., Mootha V.K., Mukherjee S., Ebert B.L., Gillette M.A. (2005). Gene set enrichment analysis: a knowledge-based approach for interpreting genome-wide expression profiles. Proc. Natl. Acad. Sci. U. S. A..

[55] Pan C., Li B., Simon M.C. (2021). Moonlighting functions of metabolic enzymes and metabolites in cancer. Mol. Cell.

[56] Hillis A.L., Lau A.N., Devoe C.X., Dayton T.L., Danai L.V., Di Vizio D. (2018). PKM2 is not required for pancreatic ductal adenocarcinoma. Cancer Metab..

[57] Lau A.N., Israelsen W.J., Roper J., Sinnamon M.J., Georgeon L., Dayton T.L. (2017). PKM2 is not required for colon cancer initiated by APC loss. Cancer Metab..

[58] Israelsen W.J., Dayton T.L., Davidson S.M., Fiske B.P., Hosios A.M., Bellinger G. (2013). PKM2 isoform-specific deletion reveals a differential Requirement for pyruvate kinase in tumor cells. Cell.

[59] Bebee T.W., Park J.W., Sheridan K.I., Warzecha C.C., Cieply B.W., Rohacek A.M. (2015). The splicing regulators Esrp1 and Esrp2 direct an epithelial splicing program essential for mammalian development. Elife.

[60] Lekva T., Berg J.P., Fougner S.L., Olstad O.K., Ueland T., Bollerslev J. (2012). Gene expression profiling Identifies ESRP1 as a potential regulator of epithelial mesenchymal transition in Somatotroph Adenomas from a Large Cohort of patients with Acromegaly. J. Clin. Endocrinol. Metab..

[61] Harvey S.E., Xu Y., Lin X., Gao X.D., Qiu Y., Ahn J. (2018). Coregulation of alternative splicing by hnRNPM and ESRP1 during EMT. RNA.

[62] Yao J., Caballero O.L., Huang Y., Lin C., Rimoldi D., Behren A. (2016). Altered expression and splicing of ESRP1 in Malignant melanoma correlates with epithelial–mesenchymal Status and tumor-associated immune Cytolytic activity. Cancer Immunol. Res..

[63] Deng G., Zhou X., Chen L., Yao Y., Li J., Zhang Y. (2020). High expression of ESRP1 regulated by circ-0005585 promotes cell colonization in ovarian cancer. Cancer Cell Int..

[64] Maciel T.T., Moura I.C., Hermine O. (2015). The role of mast cells in cancers. F1000Prime Rep..

[65] Yuen G.J., Demissie E., Pillai S. (2016). B lymphocytes and cancer: a love-hate relationship. Trends Cancer.

[66] Leontieva O.V., Ionov Y. (2009). RNA-binding motif protein 35A is a novel tumor suppressor for colorectal cancer. Cell Cycle.

[67] Fagoonee S., Picco G., Orso F., Arrigoni A., Longo D.L., Forni M. (2016). The RNA-binding protein ESRP1 promotes human colorectal cancer progression. Oncotarget.

[68] Verschueren K.H.G., Blanchet C., Felix J., Dansercoer A., De Vos D., Bloch Y. (2019). Structure of ATP citrate lyase and the origin of citrate synthase in the Krebs cycle. Nature.

[69] Fatland B.L., Ke J., Anderson M.D., Mentzen W.I., Cui L.W., Allred C.C. (2002). Molecular characterization of a Heteromeric ATP-citrate lyase that generates cytosolic acetyl-Coenzyme A in Arabidopsis. Plant Physiol..

[70] Wu T., Yu J., Gale-Day Z., Woo A., Suresh A., Hornsby M. (2020). Three essential Resources to Improve differential scanning fluorimetry (DSF) experiment. bioRxiv.

[71] Raymond M.H., Davidson A.J., Shen Y., Tudor D.R., Lucas C.D., Morioka S. (2022). Live cell tracking of macrophage efferocytosis during Drosophila embryo development in vivo. Science.

[72] Millard P., Delépine B., Guionnet M., Heuillet M., Bellvert F., Létisse F. (2019). IsoCor: isotope correction for high-resolution MS labeling experiments. Bioinformatics.

[73] Trefely S., Ashwell P., Snyder N.W. (2016). FluxFix: automatic isotopologue normalization for metabolic tracer analysis. BMC Bioinformatics.

