# Supplementary material for: ACLY alternative splicing correlates with cancer phenotypes
Source: J Biol Chem. 2024 May 28;300(7):107418. doi: 10.1016/j.jbc.2024.107418 (PMC11260853; doi:10.1016/j.jbc.2024.107418)
Supplement: Supporting Figures [file mmc1.pdf]

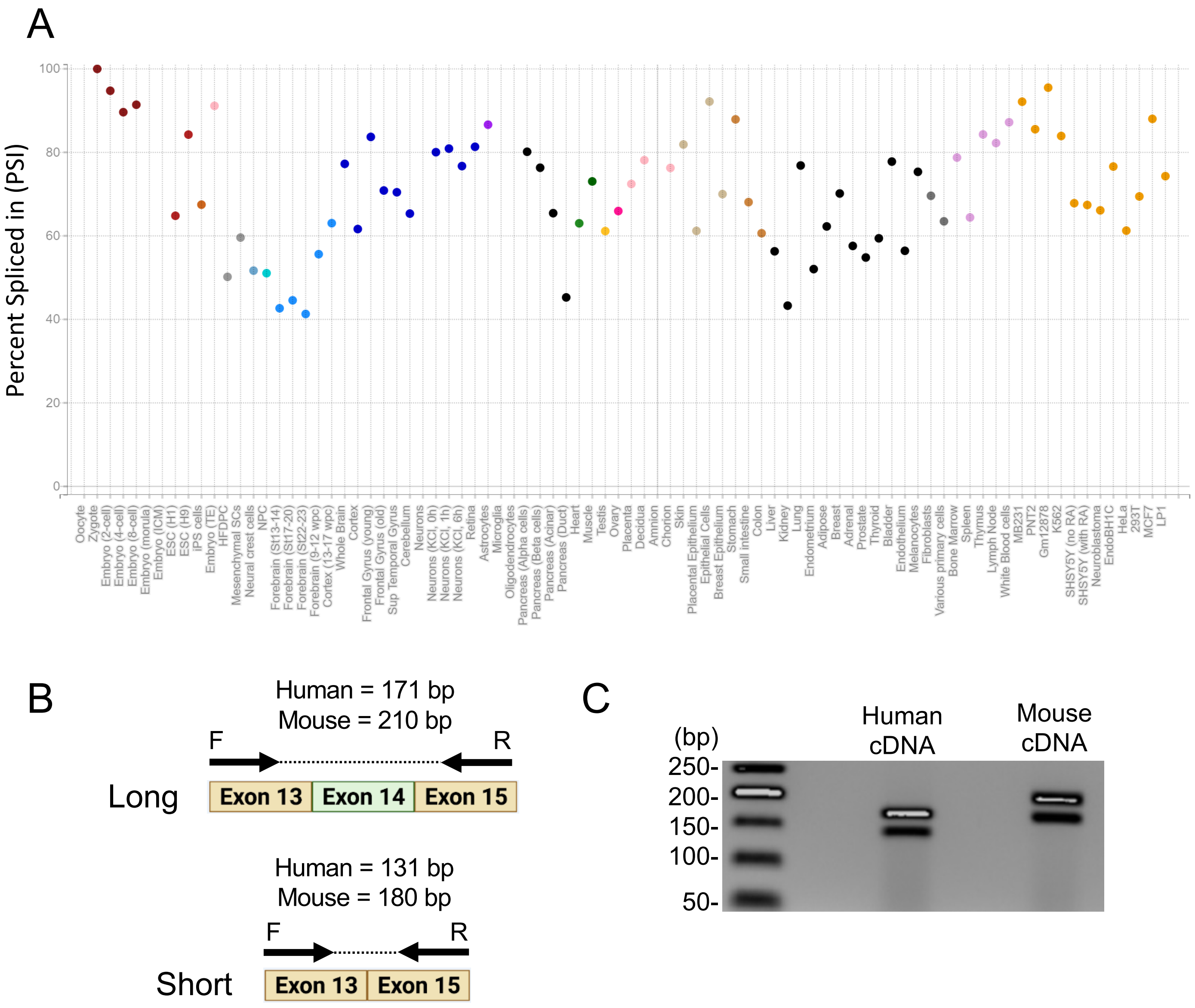

Supplemental Figure S1. A "short" ACLY isoform exists in human and mouse. (A) ACLY exon 14 PSI of several human tissues and cell lines, taken from VastDB (https://vastdb.crg.eu/gene/ENSG00000131473@hg38). (B) Schematic of RT-PCR primer design for detection of ACLY isoforms. (C) cDNA from HepG2 cells (human) and mouse liver cancer cells was amplified using the primers in (B). Exon 14 inclusion/exclusion is distinguished by migration of PCR products through an agarose gel.

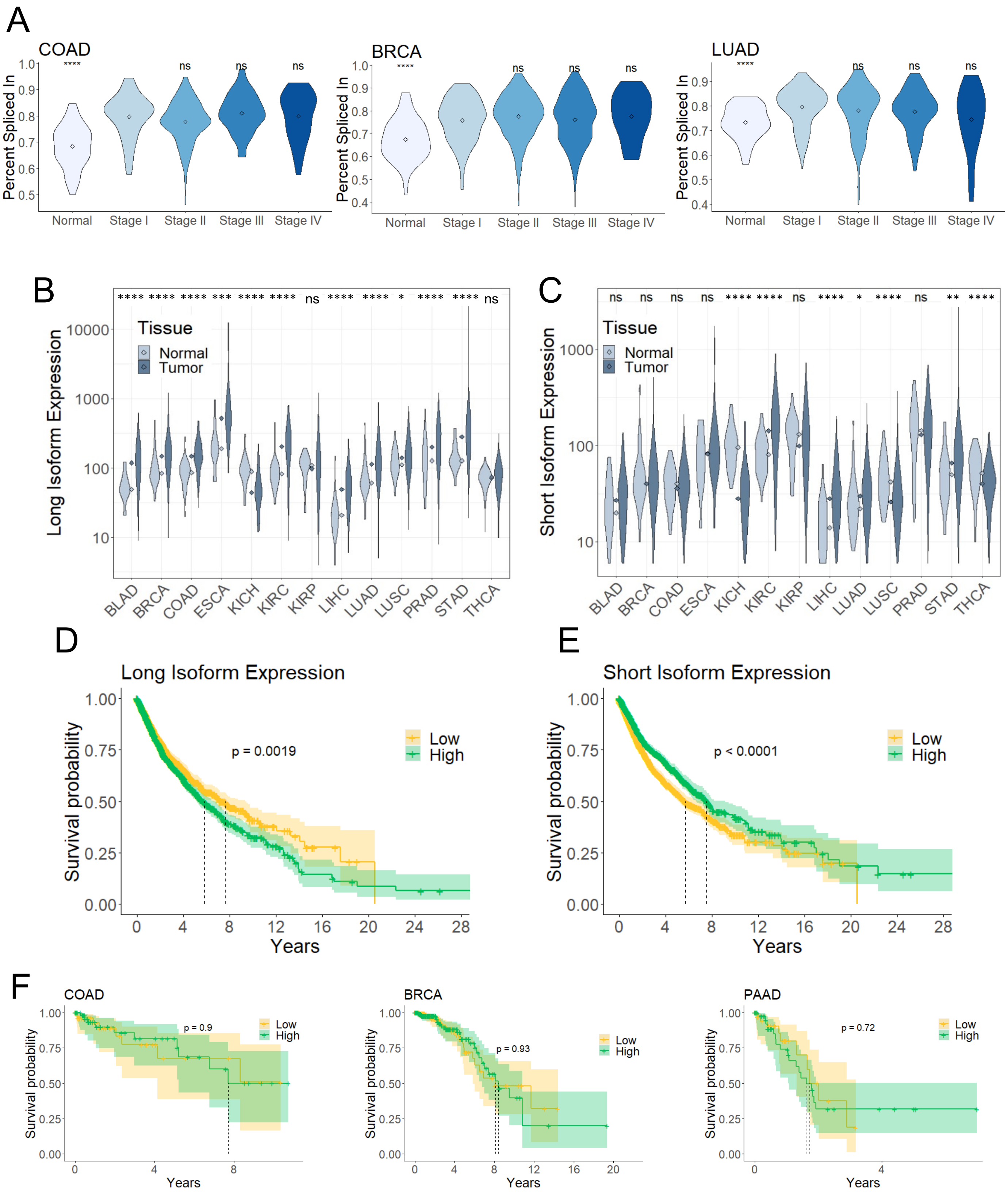

A
COAD
BRCA
LUAD
Percent Spliced In
Normal
Stage I
Stage II
Stage III
Stage IV
ns
****
B
C
Tissue
Normal
Tumor
Long Isoform Expression
Short Isoform Expression
BLAD
BRCA
COAD
ESCA
KICH
KIRC
KIRP
LIHC
LUAD
LUSC
PRAD
STAD
THCA
D
E
Long Isoform Expression
Short Isoform Expression
Survival probability
Years
Low
High
p = 0.0019
p < 0.0001
F
COAD
BRCA
PAAD
p = 0.9
p = 0.93
p = 0.72

Supplemental Figure S2. High ACLY PSI across cancers is associated with poor patient outcomes. (A) ACLY PSI by tumor stage in representative cancers: colon adenocarcinoma (COAD), breast cancer (BRCA), and lung adenocarcinoma (LUAD). Groups were compared to Stage I using two-tailed t test. Comparison of (B) long isoform and (C) short isoform expression in normal and tumor tissues. Groups were compared by two-tailed t test. Kaplan-Meyer survival curves of TCGA patients stratified by upper (high) and lower (low) quartile long ACLY (D) and short ACLY (E), where dotted lines indicated time at 0.50 survival probability. Groups were compared using log-rank test. (F) Kaplan-Meyer survival curves of TCGA patients stratified by upper (high) and lower (low) quartile ACLY PSI in representative tumor types: colon adenocarcinoma (COAD), breast cancer (BRCA), and pancreatic ductal adenocarcinoma (PAAD), where dotted lines indicated time at 0.50 survival probability. Groups were compared by log-rank test. Symbols: ns ($p > 0.05$), $p \leq 0.05$ (*), $p \leq 0.01$ (**), $p \leq 0.001$ (***), $p \leq 0.0001$ (****).

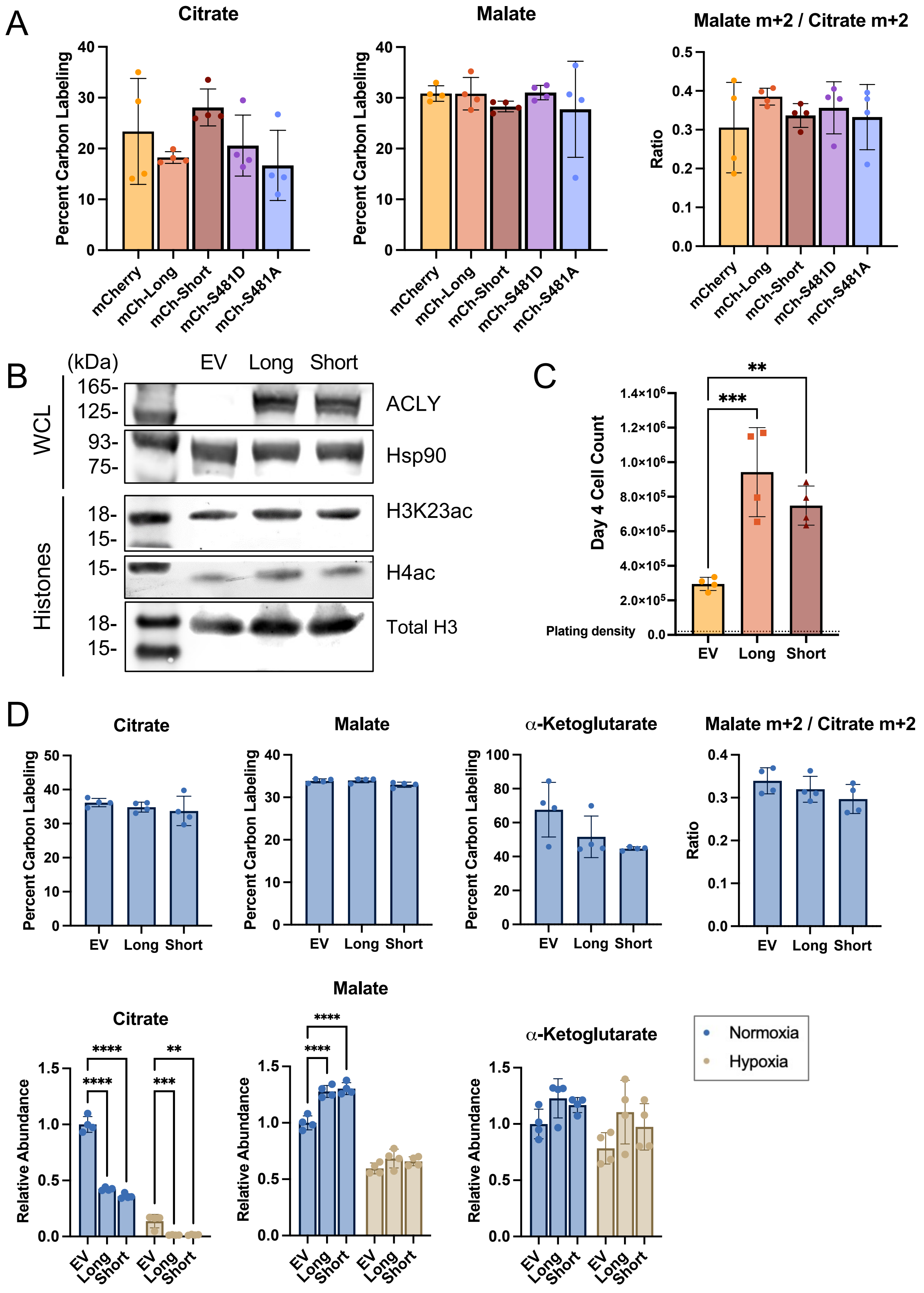
A
Citrate
Malate
Malate m+2 / Citrate m+2
Percent Carbon Labeling
Ratio
mCherry
mCh-Long
mCh-Short
mCh-S481D
mCh-S481A
B
(kDa)
EV
Long
Short
WCL
Histones
165-
125-
93-
75-
18-
15-
ACLY
Hsp90
H3K23ac
H4ac
Total H3
C
Day 4 Cell Count
Plating density
D
α-Ketoglutarate
Relative Abundance
Normoxia
Hypoxia

Supplemental Figure S3. ACLY isoforms and phosphomutants rescue function in *Acly* knockout cells. (A) ^{13}C enrichment in TCA cycle intermediates after 6h incubation with uniformly labeled ^{13}C glucose in MEFs. Differences between cell lines were not significant by one-way ANOVA. (B) Western blot of mouse hepatocellular carcinoma cells (HCCs) with *Acly* KO and human ACLY re-expression. WCL = whole cell lysate. (C) Growth of indicated HCC cell lines after 4 days in complete media. Data points represent individual plates. Cell lines were compared by one-way ANOVA. (D) ^{13}C enrichment and relative quantification of TCA cycle intermediates in HCCs after 6h incubation with uniformly labeled ^{13}C glucose at normoxia (atmospheric oxygen, blue) and hypoxia (1% O2, tan). Integrated peaks were normalized to an internal standard (1uM norvaline spike-in) and total ion count; values shown are relative to EV, normoxia. Cell lines in the same condition were compared using one-way ANOVA. Symbols: $p \leq 0.01$ (**), $p \leq 0.001$ (***), $p \leq 0.0001$ (****).

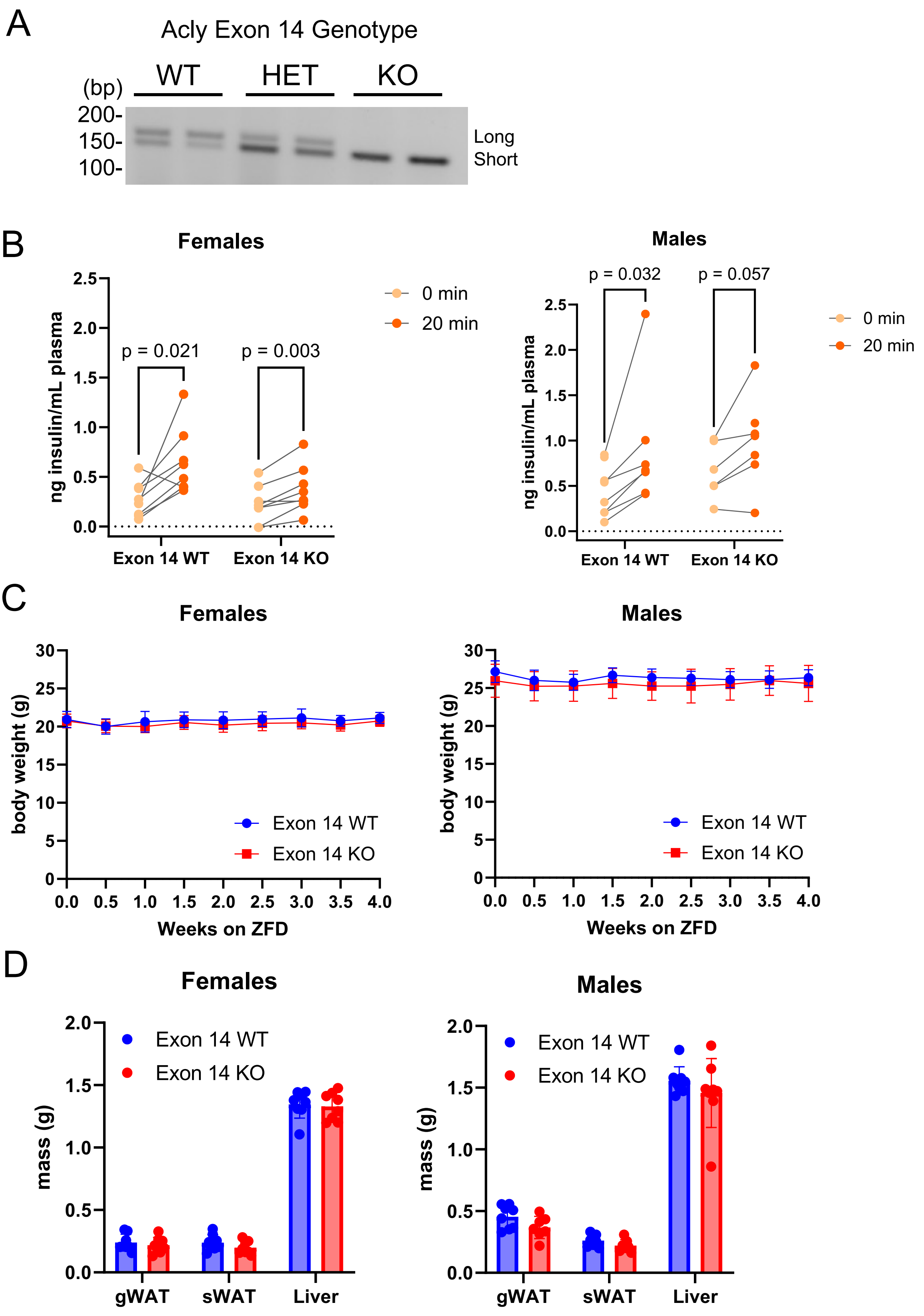
A
Acly Exon 14 Genotype
WT
HET
KO
(bp)
200-
150-
100-
Long
Short
B
Females
p = 0.021
p = 0.003
ng insulin/mL plasma
0 min
20 min
Exon 14 WT
Exon 14 KO
Males
p = 0.032
p = 0.057
C
Females
Males
body weight (g)
Weeks on ZFD
Exon 14 WT
Exon 14 KO
D
Females
Males
mass (g)
gWAT
sWAT
Liver
Exon 14 WT
Exon 14 KO

Supplemental Figure S4. ***Acly*** **exon 14 deletion in C57BL6/J mice minimally affects metabolic physiology.** (A) RT-PCR of brain tissue from one male and one female Acly exon 14 +/+, +/-, and -/- mice. (B) Plasma insulin levels during glucose tolerance test. Groups were compared by paired t test. Symbols: ns ($p > 0.05$), $p \leq 0.05$ (*), $p \leq 0.01$ (**), $p \leq 0.001$ (***), $p \leq 0.0001$ (****).

(C) Mouse body weight on ZFD. (D) Mass of indicated organs at sac after 4 weeks on ZFD. Differences between WT and KO animals were not statistically significant ($p > 0.05$) by two-tailed t test.

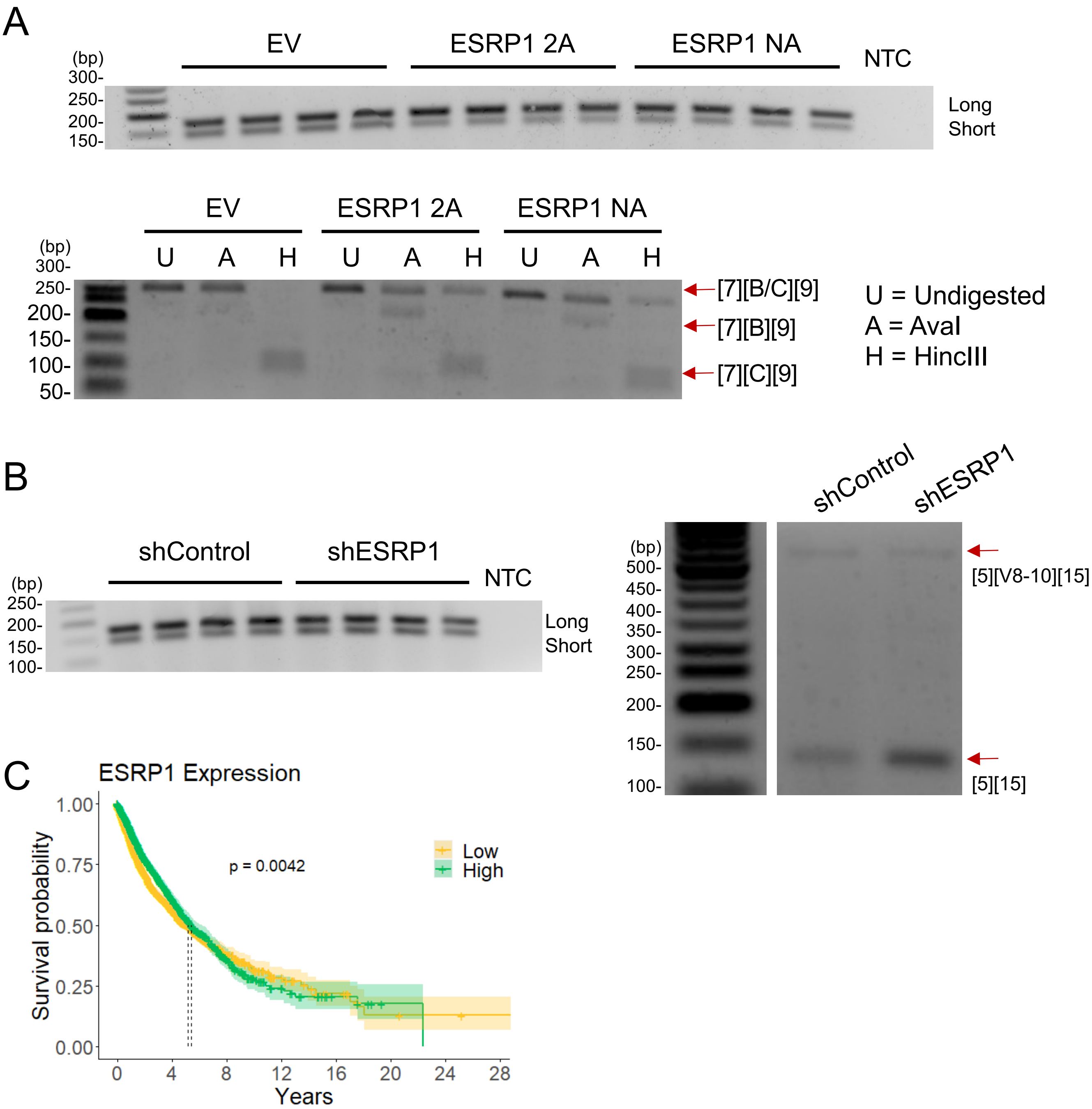

Supplemental Figure S5. ACLY is a target of the splicing factor ESRP1. (A) RT-PCR of ACLY isoforms as quantified in Figure 7D (top) and FGFR2 isoforms (bottom) in transfected HEK293T cells. NTC = no template control. Exon [B] and exon [C] of FGFR2 are approximately the same size but differ in sequence, so amplified cDNA was digested with indicated restriction endonucleases to differentiate them as described in ref 53. (B) RT-PCR of ACLY isoforms as quantified in Figure 7E (left) and CD44 isoforms (right) in transfected HCT-116 cells. NTC = no template control. (C) Kaplan-Meyer survival curve of TCGA patients stratified by upper (high) and lower (low) quartile ESRP1 expression, where dotted lines indicated time at 0.50 survival probability. Groups were compared using log-rank test.
