# Supplementary material for: ACLY alternative splicing correlates with cancer phenotypes
Source: J Biol Chem. 2024 May 28;300(7):107418. doi: 10.1016/j.jbc.2024.107418 (PMC11260853; doi:10.1016/j.jbc.2024.107418)
Supplement: Supporting Tables [file mmc2.docx]

|  |  | **ACLY PSI** | | **ESRP1** | |
| --- | --- | --- | --- | --- | --- |
| **Rank** | **Gene Set** | **NES** | **Gene Count** | **NES** | **Gene Count** |
| 1 | REACTOME_FCERI_MEDIATED_NF_KB_ACTIVATION | 2.82 | 103 | 2.65 | 99 |
| 2 | REACTOME_FCGR_ACTIVATION | 2.81 | 42 | 2.64 | 46 |
| 3 | REACTOME_FCERI_MEDIATED_MAPK_ACTIVATION | 2.79 | 56 | 2.67 | 58 |
| 4 | REACTOME_ROLE_OF_LAT2_NTAL_LAB_ON_CALCIUM_MOBILIZATION | 2.78 | 41 | 2.64 | 46 |
| 5 | REACTOME_CD22_MEDIATED_BCR_REGULATION | 2.78 | 33 | 2.63 | 38 |
| 6 | REACTOME_ROLE_OF_PHOSPHOLIPIDS_IN_PHAGOCYTOSIS | 2.78 | 55 | 2.66 | 59 |
| 7 | REACTOME_SCAVENGING_OF_HEME_FROM_PLASMA | 2.77 | 35 | 2.59 | 39 |
| 8 | REACTOME_PARASITE_INFECTION | 2.75 | 88 | 2.45 | 90 |
| 9 | REACTOME_FCERI_MEDIATED_CA_2_MOBILIZATION | 2.74 | 54 | 2.69 | 58 |
| 10 | REACTOME_ANTIGEN_ACTIVATES_B_CELL_RECEPTOR_BCR_LEADING_TO_GENERATION_OF_SECOND_MESSENGERS | 2.74 | 56 | 2.54 | 61 |
| 11 | REACTOME_CREATION_OF_C4_AND_C2_ACTIVATORS | 2.73 | 39 | 2.57 | 44 |
| 12 | REACTOME_IMMUNOREGULATORY_INTERACTIONS_BETWEEN_A_LYMPHOID_AND_A_NON_LYMPHOID_CELL | 2.71 | 107 | 2.29 | 117 |
| 13 | REACTOME_CELL_CYCLE_CHECKPOINTS | 2.68 | 242 | 2.10 | 228 |
| 14 | REACTOME_INITIAL_TRIGGERING_OF_COMPLEMENT | 2.66 | 46 | 2.51 | 52 |
| 15 | REACTOME_FORMATION_OF_THE_CORNIFIED_ENVELOPE | 2.66 | 31 | 2.59 | 36 |
| 16 | REACTOME_KERATINIZATION | 2.66 | 31 | 2.59 | 36 |
| 17 | REACTOME_FCGR3A_MEDIATE-D_IL10_SYNTHESIS | 2.66 | 65 | 2.57 | 69 |
| 18 | REACTOME_BINDING_AND_UPTAKE_OF_LIGANDS_BY_SCAVENGER_RECEPTORS | 2.64 | 60 | 2.49 | 65 |
| 19 | REACTOME_FCGAMMA_RECEPTOR_FCGR_DEPENDENT_PHAGOCYTOSIS | 2.61 | 115 | 2.32 | 117 |
| 20 | REACTOME_SIGNALING_BY_THE_B_CELL_RECEPTOR_BCR | 2.59 | 133 | 2.42 |  |
| 21 | REACTOME_CELL_CYCLE_MITOTIC | 2.56 | 478 | 1.85 | 453 |
| 22 | REACTOME_CELL_SURFACE_INTERACTIONS_AT_THE_VASCULAR_WALL | 2.56 | 133 | 2.32 | 130 |
| 23 | REACTOME_COMPLEMENT_CASCADE | 2.54 | 60 | 2.32 | 68 |
| 24 | REACTOME_FC_EPSILON_RECEPTOR_FCERI_SIGNALING | 2.52 | 151 | 2.39 | 144 |
| 25 | REACTOME_MITOTIC_SPINDLE_CHECKPOINT | 2.51 | 106 | 1.94 | 101 |
| 26 | REACTOME_DNA_REPLICATION | 2.48 | 126 | 1.86 | 116 |
| 27 | REACTOME_G2_M_CHECKPOINTS | 2.47 | 125 | 1.98 | 116 |
| 28 | REACTOME_SYNTHESIS_OF_DNA | 2.43 | 116 | 1.81 | 106 |
| 29 | REACTOME_MITOTIC_METAPHASE_AND_ANAPHASE | 2.42 | 219 | 1.71 | 208 |
| 30 | REACTOME_CHROMOSOME_MAINTENANCE | 2.39 | 84 | 1.76 | 84 |

**Supplemental Table 1. Top 30 REACTOME gene sets enriched in high ACLY PSI and high ESRP1 tumors.** Gene sets are sorted by NES in high ACLY PSI tumors. NES = normalized enrichment score. Q-value (false discovery rate) is 3.71E-09 and 7.8E-09 for all listed gene sets for ACLY PSI and ESRP1 analysis, respectively. Immune-related gene sets are shaded light gray and cell-cycle related gene sets are shaded darker gray.

|  |  | **ACLY PSI** | | **ESRP1** | |
| --- | --- | --- | --- | --- | --- |
| **Rank** | **Gene Set** | **NES** | **Q-value** | **NES** | **Q-value** |
| 1 | HALLMARK_E2F_TARGETS | 2.87 | 4.84E-10 | 2.30 | 7.1E-10 |
| 2 | HALLMARK_G2M_CHECKPOINT | 2.78 | 4.84E-10 | 2.29 | 7.1E-10 |
| 3 | HALLMARK_MYC_TARGETS_V1 | 2.32 | 4.84E-10 | 1.91 | 3.8E-06 |
| 4 | HALLMARK_ESTROGEN_RESPONSE_LATE | 2.28 | 4.84E-10 | 2.36 | 7.1E-10 |
| 5 | HALLMARK_INTERFERON_GAMMA_RESPONSE | 2.19 | 4.84E-10 | 1.44 | 2.3E-02 |
| 6 | HALLMARK_MYC_TARGETS_V2 | 2.17 | 3.14E-06 | 1.75 | 6.8E-03 |
| 7 | HALLMARK_INTERFERON_ALPHA_RESPONSE | 2.16 | 2.35E-07 | 1.66 | 7.4E-03 |
| 8 | HALLMARK_ALLOGRAFT_REJECTION | 2.13 | 6.53E-09 | N/A | N/A |
| 9 | HALLMARK_MITOTIC_SPINDLE | 1.99 | 5.64E-08 | 1.64 | 2.2E-03 |
| 10 | HALLMARK_INFLAMMATORY_RESPONSE | 1.93 | 2.11E-06 | N/A | N/A |
| 11 | HALLMARK_ESTROGEN_RESPONSE_EARLY | 1.92 | 7.94E-07 | 2.29 | 7.1E-10 |
| 12 | HALLMARK_TNFA_SIGNALING_VIA_NFKB | 1.86 | 3.16E-06 | 1.57 | 3.7E-03 |
| 13 | HALLMARK_MTORC1_SIGNALING | 1.78 | 2.88E-05 | N/A | N/A |
| 14 | HALLMARK_IL6_JAK_STAT3_SIGNALING | 1.77 | 7.14E-04 | N/A | N/A |
| 15 | HALLMARK_GLYCOLYSIS | 1.61 | 7.15E-04 | 1.64 | 1.5E-03 |
| 16 | HALLMARK_SPERMATOGENESIS | 1.60 | 4.94E-03 | N/A | N/A |
| 17 | HALLMARK_EPITHELIAL_MESENCHYMAL_TRANSITION | 1.54 | 2.44E-03 | 1.73 | 2.8E-04 |
| 18 | HALLMARK_P53_PATHWAY | 1.53 | 2.44E-03 | 1.65 | 1.3E-03 |
| 19 | HALLMARK_UNFOLDED_PROTEIN_RESPONSE | 1.48 | 1.56E-02 | N/A | N/A |
| 20 | HALLMARK_APOPTOSIS | 1.45 | 1.35E-02 | N/A | N/A |

**Supplemental Table 2. All HALLMARK gene sets enriched in high ACLY PSI and high ESRP1 tumors.** Gene sets are sorted by NES in high ACLY PSI tumors. NES = normalized enrichment score. Immune-related gene sets are shaded light gray and cell-cycle related gene sets are shaded darker gray.
